# Metal‐Coordinated NIR‐II Nanoadjuvants with Nanobody Conjugation for Potentiating Immunotherapy by Tumor Metabolism Reprogramming

**DOI:** 10.1002/advs.202404886

**Published:** 2024-07-07

**Authors:** Yeneng Dai, Ziang Guo, Dongliang Leng, Guanda Jiao, Kai Chen, Mingxuan Fu, Yang Liu, Qingming Shen, Qi Wang, Lipeng Zhu, Qi Zhao

**Affiliations:** ^1^ Cancer Centre Institute of Translational Medicine Faculty of Health Sciences University of Macau Macau SAR 999078 China; ^2^ State Key Laboratory of Organic Electronics and Information Displays & Jiangsu Key Laboratory for Biosensors Institute of Advanced Materials (IAM) Nanjing University of Posts & Telecommunications Nanjing 210023 China; ^3^ School of Life Sciences Central South University Changsha 410013 China; ^4^ MoE Frontiers Science Center for Precision Oncology University of Macau Taipa Macau SAR 999078 China

**Keywords:** amplified immunotherapy, lactate efflux, nanoadjuvants, nanobody, NIR‐II phototheranostics

## Abstract

Immune checkpoint blockade (ICB) immunotherapy remains hampered by insufficient immunogenicity and a high‐lactate immunosuppressive tumor microenvironment (TME). Herein, a nanobody‐engineered NIR‐II nanoadjuvant with targeting metabolic reprogramming capability is constructed for potentiating NIR‐II photothermal‐ferroptosis immunotherapy. Specifically, the nanoadjuvant (2DG@FS‐Nb) is prepared by metallic iron ion‐mediated coordination self‐assembly of D‐A‐D type NIR‐II molecules and loading of glycolysis inhibitor, 2‐deoxy‐D‐glucose (2DG), followed by modification with aPD‐L1 nanobody (Nb), which can effectively target the immunosuppressive TME and trigger in situ immune checkpoint blockade. The nanoadjuvants responsively release therapeutic components in the acidic TME, enabling the precise tumor location by NIR‐II fluorescence/photoacoustic imaging while initiating NIR‐II photothermal‐ferroptosis therapy. The remarkable NIR‐II photothermal efficiency and elevated glutathione (GSH) depletion further sensitize ferroptosis to induce severe lipid peroxidation, provoking robust immunogenic cell death (ICD) to trigger anti‐tumor immune response. Importantly, the released 2DG markedly inhibits lactate generation through glycolysis obstruction. Decreased lactate efflux remodels the immunosuppressive TME by suppressing M2 macrophage proliferation and downregulating regulatory T cell levels. This work provides a new paradigm for the integration of NIR‐II phototheranostics and lactate metabolism regulation into a single nanoplatform for amplified anti‐tumor immunotherapy combined with ICB therapy.

## Graphical abstract

NIR‐II nanoadjuvants with metabolic reprogramming capability is constructed by metallic iron ion‐mediated coordination self‐assembly. After nanobody‐mediated active targeting and evoking immune checkpoint blockade, nanoadjuvants responsively decompose triggered by acidic tumor microenvironment (TME) and enable synergistic NIR‐II phototheranostics and ferroptosis, provoking anti‐tumor immune responses. The remodeling of the immunosuppressive TME through lactate metabolism regulation further potentiates the efficiency of immunotherapy.

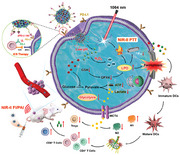

## 1 Introduction

Immune checkpoint blockade (ICB) therapy, such as the blockade of the programmed cell death protein 1/programmed cell death ligand 1 (PD‐1/PD‐L1) axis, has achieved unprecedented clinical benefits.^[^ ^1^ ^]^ However, single ICB therapy dependent only on reactivation of already existing autologous cytotoxic T lymphocytes (CTLs) cannot achieve satisfactory anti‐tumor immunotherapeutic efficacy because of inadequate infiltration of CTLs into tumor tissues.^[^ ^2^ ^]^ More crucially, blocking antibodies are also easily cleared from the body because of nonspecific accumulation induced by systemic administration, leading to low blockade efficiency.^[^ ^3^ ^]^ Therefore, it is desirable to construct an antibody deliver system to delivery blockade antibodies into tumor tissues and achieve efficient immune outcomes by combining ICB therapy with other treatment modalities.

Phototheranostics has attracted much attention because of its ability to induce localization and eradication of tumors by photon excitation through noninvasive and spatiotemporally controllable means.^[^ ^4^ ^]^ Of particular interest is phototheranostics implemented by second near‐infrared light (NIR‐II, 1000–1700 nm), showing irreplaceable merits over NIR‐I light (650–900 nm) owing to the dramatically reduced light‐tissue interactions in this wavelength range.^[^ ^5^ ^]^ For example, NIR‐II photothermal therapy (PTT) can convert photoenergy into hyperthermia for tumor ablation with higher tissue penetration depth and maximum permissible exposure (MPE). Moreover, NIR‐II fluorescence imaging (FI) and photoacoustic imaging (PAI) enable precise positioning of deep/hidden tumors with higher signal‐to‐background ratio (SBR) and imaging resolution.^[^ ^6^ ^]^ Ferroptosis is a newly discovered non‐apoptotic form of programmed cell death, which is induced by iron‐dependent accumulation of lethal lipid peroxidation (LPO) through the iron catalytic Fenton reaction, accompanied by reduced production of glutathione peroxidase 4 (GPX4).^[^ ^7^ ^]^ The severe LPO will perturb cellular redox homeostasis and trigger the rupture of the outer membrane and eventually cell death.^[^ ^8^ ^]^ Recently, the combination of catalytically active iron ions and NIR‐II phototheranostics has gained considerable attention.^[^ ^9^ ^]^ More importantly, NIR light‐activated synergistic therapy can trigger immunogenic cell death (ICD) through the release of damage‐associated molecular patterns (DAMPs), provoking a strong anti‐tumor immune response by promoting the maturation of dendritic cells (DCs) and recruiting more antigen‐specific CTLs.^[^ ^10^ ^]^ This immune activation mode is expected to compensate for the insufficient infiltration of CTLs in ICB therapy.

Despite remarkable success, immunotherapy progress is still hindered by the immunosuppressive tumor microenvironment (TME) caused by metabolic reprogramming of tumor cells.^[^ ^11^ ^]^ Tumor cells metabolize glucose for energy supply through glycolysis, which is termed the “Warburg effect.”^[^ ^12^ ^]^ As an important by‐product of glycolytic metabolism, lactate (LA) will flow out of tumor cells through monocarboxylate transporter 4 (MCT4) mediated LA efflux. Excessive LA accumulated in the TME can not only be used as an energy source to stimulate tumor angiogenesis, invasion, and metastasis, but also promote tumor immune escape by inducing the transformation of tumor‐associated macrophage (TAM) phenotype from the anti‐tumor M1 type to the pro‐tumor M2 type while diminishing effector T cell proliferation.^[^ ^13^ ^]^ Furthermore, recent studies have indicated that regulatory T cells (Tregs) combat effector T cells under high‐LA conditions provided by tumor glycolysis metabolism.^[^ ^13^, ^14^ ^]^ Briefly, the modulation of LA metabolism has emerged as a novel strategy to reverse and reshape the immunosuppressive TME, thereby sensitizing anti‐tumor immune responses.^[^ ^15^ ^]^

**Scheme 1:**
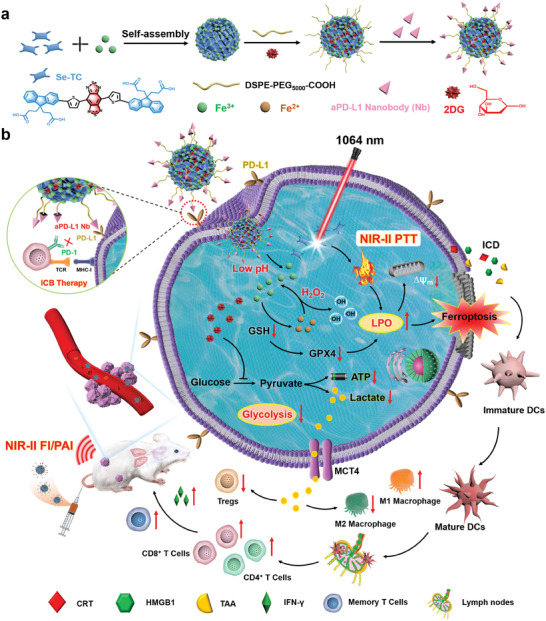
Schematic illustration of 2DG@FS‐Nb for potentiating anti‐tumor immunotherapy via lactate (LA) metabolic reprogramming. a) Preparation of aPD‐L1 nanobody‐modified NIR‐II nanoadjuvants 2DG@FS‐Nb. b) NIR‐II imaging‐guided ICB therapy and NIR‐II PTT‐ ferroptosis synergistically evoke an amplified immune response together with LA metabolism regulation.

In view of the above‐mentioned main challenges, herein, an innovative LA inhibition strategy is proposed in combination with ICB therapy for synergistically amplified immunotherapy. The immunomodulatory nanoadjuvant (2DG@FS‐Nb) was constructed through coordinated self‐assembly of the D‐A‐D type NIR‐II molecule Se‐TC with iron ions Fe(III), followed by loading with the glycolysis inhibitor 2‐deoxy‐D‐glucose (2DG) and modification with aPD‐L1 nanobody (Nb). After Nb‐mediated active targeting and evoking in situ ICB toward tumor cells, acidic conditions in the TME can trigger the decomposition of 2DG@FS‐Nb, releasing Se‐TC for NIR‐II FI/PAI‐guided NIR‐II PTT (**Scheme** **1**). The released Fe(III) can catalyze endogenous hydrogen peroxide (H_2_O_2_) to generate hydroxyl radicals (∙OH) for potent ferroptosis by LPO. The depletion of glutathione (GSH) in the catalytic cycle and the NIR‐II photothermal effect can further sensitize ferroptosis. Moreover, NIR‐II light excited synergistic therapy can induce remarkable ICD and promote DC maturation, provoking a cascade of anti‐tumor immune responses combined with ICB therapy by Nb. Particularly, the release of 2DG inhibits LA production by regulating glycolytic metabolism in tumor cells, thus reversing the immunosuppressive TME and enhancing anti‐tumor immunity efficiency. In this study, a new paradigm was proposed for constructing NIR‐II self‐assembly nanoadjuvants and amplifying efficiency of immunotherapy through tumor metabolism reprogramming.

## 2 Results and Discussion

### 2.1 Preparation and Characterization of 2DG@FS‐Nb

Carboxyl‐functionalized D‐A‐D type NIR‐II organic small molecule Se‐TC was first synthesized based on previously reported literature,^[^ ^16^ ^]^ in which [1,2,5]selenadiazolo[3,4‐f]benzo[c][1,2,5]thiadiazole was selected as the electron acceptor unit, and thiophene and fluorene were chosen as the electron donor units (Scheme S2, Supporting Information). The structure of Se‐TC was verified by ^1^H NMR and MALDI‐TOF mass spectrometry (Figures S1 and S2, Supporting Information). Density functional theory (DFT) calculations were performed to explore the photophysical properties of Se‐TC. The S_0_ optimized geometry displayed a small dihedral angle of less than 20° and revealed a planar conjugated skeleton (Figure S3a, Supporting Information). The planar molecular structure is favorable for long‐wavelength absorption, as evidenced by the narrow band gap between molecular orbitals (Figure S3b, Supporting Information). The electrostatic potential map (ESP) indicated that the terminal carboxyl groups of Se‐TC could easily coordinate with metal ions to form self‐assembled nanostructures (Figure S4, Supporting Information). The optical properties of Se‐TC were further detected on the basis of theoretical calculations. As shown in Figure S5 (Supporting Information), a broad absorption range from 500 to 1100 nm was displayed in the absorption spectrum of Se‐TC in THF, as well as a strong absorption peak at ≈920 nm. Moreover, excited by 808 nm laser, Se‐TC exhibited significant fluorescence emission extending to 1400 nm with a peak at 1100 nm.

**Figure 1:**
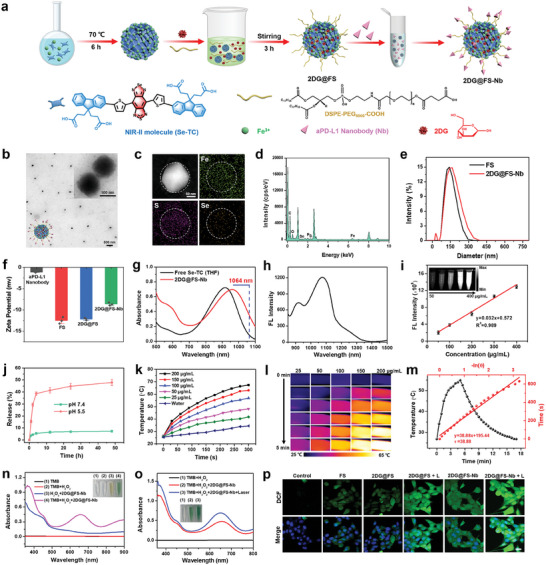
Preparation and characterization of NIR‐II self‐assembly nanoadjuvants. a) Chemical structure of NIR‐II molecules and the assembly procedure of 2DG@FS‐Nb. b) Transmission electron microscopy image, c) elemental mapping images, and d) energy‐dispersive spectroscopy (EDS) analysis of 2DG@FS‐Nb. e) Size distributions of FS and 2DG@FS‐Nb. f) Zeta potential of each component. g) UV–vis absorption spectra, h) fluorescence emission spectrum, and i) concentration‐dependent NIR‐II fluorescence images of 2DG@FS‐Nb. j) Percentage of 2DG released from 2DG@FS‐Nb at different pH values. k) Temperature changes and l) infrared thermal images of 2DG@FS‐Nb aqueous solution under 1064 nm laser irradiation. m) Temperature changes and linear regression data analysis during a heating‐cooling cycle. n,o) Absorption spectra of TMB with different treatments. p) Fluorescence images of DCFH‐DA in 4T1 cells with various treatments. Scale bar: 20 µm.

The sequence and preparation process of aPD‐L1 nanobody (Nb) were depicted in Scheme S1 (Supporting Information). The NIR‐II nanohybrid was prepared through Fe(III)‐driven coordination self‐assembly of Se‐TC, which showed a uniformly dispersed spherical morphology in THF (Figure S6, Supporting Information). To obtain water‐soluble nanoadjuvants with Nb modification, the glycolysis inhibitor 2DG was loaded into the self‐assembled nanostructure, followed by modification with long‐chain PEG and coupling with aPD‐L1 nanobody to form an NIR‐II immunomodulatory nanoadjuvant (2DG@FS‐Nb) with tumor targeting and metabolic regulation capabilities (**Figure** **1**a). The transmission electron microscopy (TEM) images clearly showed a spherical structure of 2DG@FS‐Nb (Figure 1b). Elemental mapping images and energy dispersive X‐ray spectroscopy (EDS) analysis suggested the symmetrical distribution of elemental iron, sulfur, and selenium in 2DG@FS‐Nb (Figure 1c,d), implying the successful preparation of NIR‐II self‐assembly nanoadjuvants. After 2DG loading and Nb modification, the diameter of 2DG@FS‐Nb increased from 142 nm (FS NPs) to 164 nm (Figure 1e). Additionally, 2DG@FS‐Nb displayed a negative zeta potential, which was ascribed to the modification of PEG with carboxyl groups and the conjugation of Nb with a negative charge (Figure 1f). Moreover, the 2DG@FS‐Nb lane exhibited obvious protein retardation caused by the high molecular weight through sodium dodecyl sulfate‐polyacrylamide gel electrophoresis (SDS‐PAGE) analysis (Figure S7, Supporting Information), verifying the successful conjugation of Nb on the surface of 2DG@FS. aPD‐L1 nanobody and 2DG@FS‐Nb both exhibited high binding activity toward PD‐L1 antigen (Figure S8, Supporting Information), indicating that antibody conjugation does not change the antigen binding capacity of the nanobody. Notably, the absorption of 2DG@FS‐Nb exhibited a visible red shift caused by molecular aggregation compared to free Se‐TC, as well as a significant absorption at 1064 nm (Figure 1g). Moreover, 2DG@FS‐Nb emitted prominent fluorescence emission from 900 to 1400 nm under 808 nm laser excitation (Figure 1h). Figure 1i showed concentration‐dependent NIR‐II fluorescence images, confirming the great potential of 2DG@FS‐Nb for the precise diagnosis and treatment of deep‐seated tumors by NIR‐II phototheranostics. Next, the drug release of 2DG@FS‐Nb under acidic conditions was investigated. Compared to that at pH 7.4, a rapid release of 2DG from 2DG@FS‐Nb was observed at pH 5.5 (Figure 1j). The disassembly of the nanoadjuvant initiated by acidic conditions was confirmed by TEM images, which were accompanied by significant size changes of the nanoparticles (Figure S9, Supporting Information).

### 2.2 NIR‐II Photothermal Effects and Reactive Oxygen Species (ROS) Production In Vitro

Ascribed to the prominent NIR‐II light harvesting capability by absorption spectra, we evaluated the NIR‐II photothermal performance of the self‐assembly nanoadjuvant under 1064 nm laser irradiation (1 W/cm^2^). As shown in Figure 1k, 2DG@FS‐Nb exhibited rapid temperature lifting with increased concentrations and time, and the maximum temperature reached 67.4 °C at a high concentration of 200 µg mL^−1^, as evidenced by the concentration‐ and time‐dependent enhancement of the infrared thermal images (Figure 1l). The adjustment of the laser power density (from 0.5 to 1.5 W cm^−2^) can significantly affect the temperature changes of the solution (Figure S10a, Supporting Information). Repeated heating/cooling cycles exhibited negligible decay of the maximal temperature via successive laser on/off cycles (Figure S10b, Supporting Information), illustrating the good photothermal stability of 2DG@FS‐Nb. The photothermal conversion efficiency (*η*) of ≈30.15% calculated from a heating/cooling cycle (Figure 1m), further confirmed that 2DG@FS‐Nb can serve as an effective NIR‐II photothermal agent for tumor ablation. Ascribed to excellent light absorption ability of 2DG@FS‐Nb, photoacoustic images of 2DG@FS‐Nb solution excited by 1064 nm laser exhibited concentration‐dependent enhancement of the photoacoustic signal (Figure S11, Supporting Information).

To explore the ability of 2DG@FS‐Nb catalyzing H_2_O_2_ to produce ∙OH through Fe(III) mediated Fenton reaction, TMB was selected as the capture probe, which can be oxidized by ∙OH to blue oxTMB. As indicated in Figure 1n, TMB remained colorless without obvious absorption bands after the addition of H_2_O_2_, indicating that H_2_O_2_ cannot drive the oxidation of TMB. However, the addition of 2DG@FS‐Nb turned the colorless solution blue in the presence of H_2_O_2_, as evidenced by a significant absorption peak at ≈652 nm, illustrating the Fenton activity of 2DG@FS‐Nb. Furthermore, the solution of TMB and 2DG@FS‐Nb exhibited a gradient color change after adding different concentrations of H_2_O_2_, which was verified by the increased absorptions (Figure S12, Supporting Information). The influence of NIR‐II photothermal performance on ∙OH generation was further explored by 1064 nm laser irradiation on a solution of TMB and H_2_O_2_ treated with 2DG@FS‐Nb. As shown in Figure 1o, NIR‐II laser irradiation significantly increased the oxidation of TMB, with a higher absorption band compared to that without laser irradiation. This result confirms that NIR‐II photothermal effect‐induced hyperthermia can enhance the Fenton activity of 2DG@FS‐Nb and promote ∙OH generation through photothermally promoted ionization. We further evaluated the generation of ROS induced by 2DG@FS‐Nb at the cellular level. Obvious green fluorescence of DCF was presented in 4T1 cells treated with FS and 2DG@FS, indicating Fe(III)‐induced intracellular ROS production (Figure 1p). In contrast, 2DG@FS‐Nb‐treated cells exhibited brighter fluorescence, enhancing by an ≈2.72‐fold compared to the 2DG@FS‐treated group (Figure S13, Supporting Information), possibly attributed to the improved intracellular uptake of 2DG@FS‐Nb through Nb‐mediated positive targeting. Notably, 2DG@FS‐Nb‐treated group exhibited the brightest fluorescence under 1064 nm laser irradiation, with an ≈1.73‐fold enhancement compared to that of the group without laser irradiation. These results confirm that 2DG@FS‐Nb can be used as an inducer of ROS to produce strong oxidative stress under NIR‐II laser irradiation, promoting high concentrations of LPO and inducing efficient ferroptosis.

### 2.3 Cellular Targeted Uptake, GSH Depletion, Lipid Peroxidation, and Mitochondrial Depolarization

**Figure 2:**
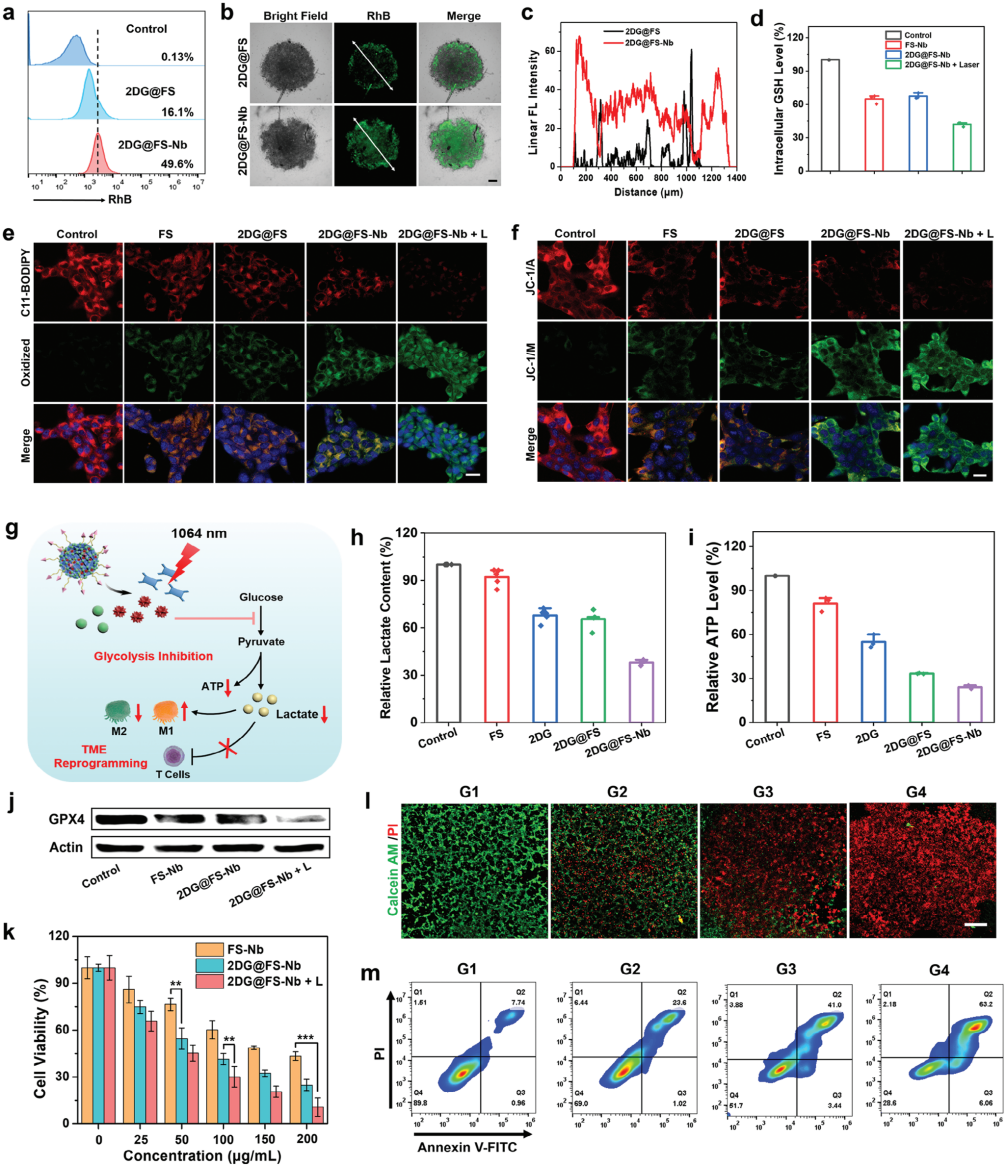
Cellular uptake, LPO, glycolysis inhibition, and cytotoxicity analysis. a) Flow cytometry analysis of cellular uptake, b) CLSM images and c) surface plot image of 4T1 3D cell spheroids treated with 2DG@FS and 2DG@FS‐Nb. Scale bar: 200 µm. d) GSH content and j) GPX4 expression induced by various treatments. e) LPO and f) MMP of 4T1 cells with various treatments using the C11‐BODIPY and JC‐1 probes, respectively. Scale bar: 20 µm. g) Schematic illustration of 2DG@FS‐Nb‐mediated glycolysis inhibition and TME reprogramming. h) Extracellular LA content and i) intracellular ATP level of 4T1 cells. k) Viability assays of 4T1 cells treated with FS‐Nb and 2DG@FS‐Nb with or without 1064 nm laser irradiation. l) Live/dead cell staining and m) flow cytometry analysis of 4T1 cells undergoing the following treatments: G1: Control, G2: FS‐Nb, G3: 2DG@FS‐Nb, and G4: 2DG@FS‐Nb + Laser. Scale bar: 500 µm.

Due to the excellent optical performance of Rhodamine B (RhB) (Figure S14, Supporting Information), RhB‐doped 2DG@FS and 2DG@FS‐Nb were added to 4T1 cells for the evaluation of Nb‐mediated positive targeting ability by flow cytometry. The results revealed that 4T1 cells treated with 2DG@FS‐Nb exhibited remarkable fluorescence of RhB, and the uptake rate was 3.08‐fold higher than that of the group treated with 2DG@FS (**Figure** **2**a). Intracellular fluorescence images showed the time‐dependent cellular uptake of 2DG@FS‐Nb (Figure S15, Supporting Information). After 4 h of incubation, 2DG@FS‐Nb gradually migrated to the cytoplasm, as evidenced by enhanced fluorescence in the periphery of the nucleus. In 3D cell spheroid uptake experiment, 2DG@FS‐Nb group displayed more green fluorescence diffusing into the spheroids compared with the 2DG@FS group, with a deeper penetration depth within cell spheroid (Figure 2b,c; Figure S16, Supporting Information). These results indicate improved intracellular uptake of 2DG@FS‐Nb through active recognition and binding of Nb toward PD‐L1 on the surface of tumor cells. Overexpressed GSH in tumor cells is a ROS‐scavenging antioxidant and a co‐factor of GPX4‐catalyzed lipid repair. Therefore, GSH depletion plays an important role in enhancing LPO and boosting ferroptosis. The level of GSH in 4T1 cells treated with FS‐Nb and 2DG@FS‐Nb was markedly decreased (Figure 2d), which was attributed to the reduction reaction between Fe(III) and GSH. Moreover, the GSH content was further reduced after treatment with 2DG@FS‐Nb plus 1064 nm laser irradiation, with a 1.66‐fold reduction compared to the 2DG@FS‐Nb‐treated group. The partial hyperthermia induced by NIR‐II laser irradiation can promote GSH‐mediated reduction of Fe(III) to Fe(II) and production of glutathione disulfide (GSSG), which could further inhibit the expression of GPX4. As shown in Figure 2j, cells treated with 2DG@FS‐Nb plus laser irradiation exhibited the lowest expression of GPX4 by western blotting analysis. Obvious immunofluorescence attenuation of GPX4 was observed in the groups treated with FS‐Nb and 2DG@FS‐Nb (Figure S17a, Supporting Information). Notably, 2DG@FS‐Nb‐treated cells exhibited the weakest fluorescence of GPX4 under 1064 nm laser irradiation, decreasing by 1.86‐fold compared to the 2DG@FS‐Nb‐treated group (Figure S17b, Supporting Information). Inactivation of GPX4 inhibits lipid repair in tumor cells by blocking the conversion of toxic LPO to nontoxic hydroxyl compounds (LOH). Therefore, the LPO levels in 4T1 cells were evaluated after various treatments using C11‐BODIPY as a ratiometric fluorescent indicator, which emits green fluorescence upon oxidation. The cells treated with FS, 2DG@FS, and 2DG@FS‐Nb manifested obvious green fluorescence, which was accompanied by attenuated red fluorescence (Figure 2e), indicating the production of LPO. Notably, the 2DG@FS‐Nb plus laser irradiation group exhibited the brightest green fluorescence and the darkest red fluorescence. These results verified the augmented accumulation of LPO through enhanced GPX4 inactivation and ROS production under NIR‐II laser irradiation, thus facilitating ferroptosis of tumor cells.

Ferroptosis‐induced intracellular oxidative stress may lead to mitochondrial dysfunction, which is accompanied by the loss of mitochondrial membrane potential (MMP) and mitochondrial shrinkage. Mitochondrial damage was evaluated by the detection of MMP using the JC‐1 probe, which emits red fluorescence as aggregates at high MMP but presents green fluorescence as monomers at relatively low MMP. As shown in Figure 2f, compared to the dense red fluorescence representing intact MMP in the control group, 4T1 cells treated with FS, 2DG@FS and 2DG@FS‐Nb exhibited distinct green fluorescence, indicating mitochondrial depolarization due to decreased MMP. Additionally, a further decline of MMP was demonstrated by the treatment with 2DG@FS‐Nb plus 1064 nm laser irradiation, implying aggravated mitochondrial abnormality due to enhanced oxidative stress by NIR‐II laser irradiation.

### 2.4 Intracellular Glycolysis Inhibition

2DG, a glucose derivative, has been confirmed to induce severe tumor starvation by blocking the generation of glycolytic adenosine 5′‐triphosphate (ATP) and LA.^[^ ^12^, ^17^ ^]^ The decreased LA efflux by glycolysis metabolism blockade further reverse and reprogram the immunosuppressive TME (Figure 2g). As characteristic metabolites of glycolysis, the content of LA and ATP were detected after various treatments. As indicated in Figure 2h,i, the extracellular LA content and intracellular ATP level were significantly reduced in the groups treated with 2DG and 2DG@FS after 24 h of incubation, indicating the glycolysis inhibition induced by 2DG. A further decline in LA and ATP levels was observed in the group treated with 2DG@FS‐Nb, with a reduction of ≈61.8 and 74.3%, respectively, compared to the control group, which was attributed to enhanced endocytosis by Nb‐mediated positive targeting. Moreover, the extracellular LA content gradually decreased with increasing 2DG concentrations within 2DG@FS‐Nb (Figure S18, Supporting Information), illustrating a concentration‐dependent LA inhibition effect. These results confirm that 2DG@FS‐Nb can effectively hinder LA production by inhibiting cancer cellular glycolysis, forming a TME with low levels of LA.

### 2.5 In Vitro Cytotoxicity Analysis

We next evaluated the synergistic therapeutic effect of 2DG@FS‐Nb on tumor cells using the CCK‐8 assay. As indicated in Figure 2k, a moderate antiproliferative effect was observed in 4T1 cells treated with FS‐Nb, with a cell survival rate of ≈43.39% at the high concentration, due to the therapeutic effect of Fe(III)‐induced ferroptosis. Additionally, 2DG@FS‐Nb presented higher cytotoxicity, which was attributed to the inhibition of the 2DG‐mediated glycolytic metabolic pathway. In particular, 2DG@FS‐Nb displayed a prominent cell‐killing effect under NIR‐II laser irradiation, with a cell viability of ≈10.75%, ascribed to the synergistic contribution of glycolysis inhibition and NIR‐II photothermal amplified ferroptosis. Cytotoxicity was visualized by live/dead cell staining. Compared to the control and FS‐Nb‐treated groups, more red fluorescence representing dead cells, accompanied by reduced green fluorescence of live cells, was observed in 2DG@FS‐Nb‐treated cells with or without 1064 nm laser irradiation (Figure 2l), indicating the synergistically therapeutic effect of NIR‐II PTT‐ferroptosis along with glycolysis inhibition. Further quantitation of flow cytometry revealed the highest cell apoptosis ratio of ≈71.4% in the 2DG@FS‐Nb‐treated group under 1064 nm laser irradiation (Figure 2m), compared to the other treatment groups. These results confirm that 2DG@FS‐Nb can effectively inhibit tumor cell growth through synergistic therapy of ferroptosis, NIR‐II PTT, and glycolysis inhibition.

### 2.6 ICD Effect, BMDC Activation, and Macrophage Polarization In Vitro

**Figure 3:**
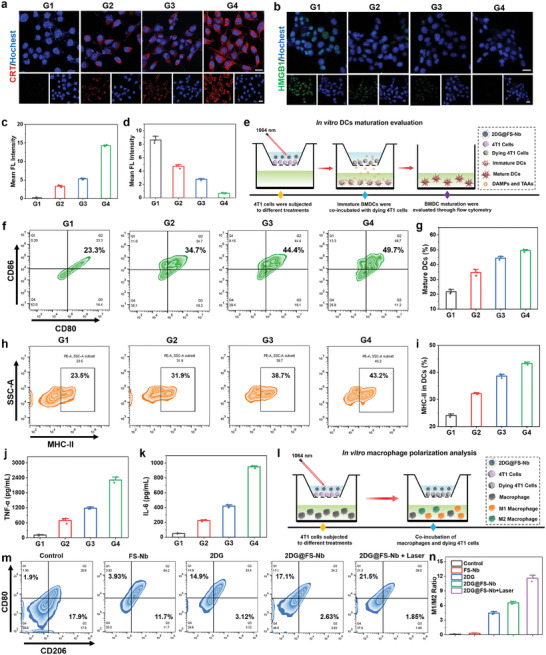
ICD effect, BMDC activation, and macrophage polarization in vitro. a,b) Immunofluorescence staining images and c,d) corresponding fluorescence intensity analysis of CRT exposure and HMGB1 release. Scale bar: 20 µm. e) Schematic diagram of Transwell experiments for BMDC maturation. f) Flow cytometric plots and g) statistical data for DC maturation gated by CD11c^+^ cells. h) Flow cytometric plots and i) statistical data of MHC‐II expression on the surface of DCs. Detection of j) TNF‐*α* and k) IL‐6 in the cell medium. l) Schematic diagram of Transwell systems for macrophage polarization in vitro. m) Flow cytometric plots and n) M1/M2 ratio analysis of macrophage differentiation after different treatments, CD80^+^CD206^−^ for M1 macrophages and CD80^−^CD206^+^ for M2 macrophages. G1: Control, G2: FS‐Nb, G3: 2DG@FS‐Nb, G4: 2DG@FS‐Nb + Laser.

Through the synergistic therapy of glycolysis inhibition and NIR‐II PTT‐ferroptosis, 2DG@FS‐Nb not only trigger tumor cell apoptosis but also provoke anti‐tumor immune responses by inducing the ICD effect, which is accompanied by releasing DAMPs. This promotes the maturation of antigen‐presenting cells, thereby recruiting and activating more cytotoxic T lymphocytes. As a marker of ICD, calreticulin (CRT) from the endoplasmic reticulum is transferred to the surface of tumor cells under stress stimulus, followed by binding to the membrane surface of DCs to stimulate DC maturation. Surface exposure of CRT was detected in 4T1 cells after various treatments. The immunofluorescence staining revealed the evident red fluorescence of CRT on the surface of 4T1 cells treated with FS‐Nb and 2DG@FS‐Nb (**Figure** **3**a), indicating distinct surface exposure of CRT due to the synergistic effect of ferroptosis and glycolysis obstruction. Notably, the 2DG@FS‐Nb plus laser irradiation treated group exhibited brighter CRT fluorescence, which was enhanced by ≈2.79‐fold compared to the 2DG@FS‐Nb treated group (Figure 3c), indicating that the therapeutic effect of NIR‐II PTT can facilitate the release of CRT. Another important ICD marker, high mobility group box 1 (HMGB1), was released from the nucleus to the extracellular matrix upon apoptotic stress, triggering the secretion of pro‐inflammatory factors from mature DCs. The most severe fluorescence decay occurred in the nucleus treated with 2DG@FS‐Nb plus laser irradiation (Figure 3b), with an ≈12.66‐fold decrease compared to the control group (Figure 3d), suggesting the most dominant HMGB1 release from the nucleus. These results confirm that 2DG@FS‐Nb can effectively elicit ICD effect and subsequent immune responses by NIR‐II laser irradiation.

DC maturation plays an important role in initiating and regulating anti‐tumor immune responses, which is attributed to the fact that mature DCs can activate cytotoxic T lymphocytes through antigen cross‐presentation, accompanied by the secretion of pro‐inflammatory cytokines. To evaluate DC maturation induced by 2DG@FS‐Nb‐mediated synergistic therapy, 4T1 cells subjected to various treatments and bone‐marrow‐derived DCs (BMDCs) were co‐cultured for 24 h in a Transwell system, followed by flow cytometry analysis of the costimulatory molecules CD80 and CD86 (Figure 3e). As shown in Figure 3f and Figure S19 (Supporting Information), FS‐Nb treatment can trigger distinct maturation of DCs, which is ascribed to the ICD evoked by ferroptosis. Importantly, 2DG@FS‐Nb plus 1064 nm laser irradiation led to the highest rates of DC maturation (Figure 3g), with 2.13‐ and 1.43‐fold increases in the maturation percentage compared to the control group and FS‐Nb treated group, respectively, and owing to the ICD effect elicited by NIR‐II PTT amplified ferroptosis together with metabolic reprogramming. Additionally, the expression level of major histocompatibility complex II (MHC‐II) on the surface of DCs was evaluated after various treatments. The progressively improved expression of MHC‐II was observed in the treatment groups (Figure 3h). The group treated with 2DG@FS‐Nb plus 1064 nm laser irradiation exhibited the highest expression level of MHC‐II on the surface of DCs, increasing by ≈1.84‐fold compared to the control group (Figure 3i). This will further enhance the recognition and binding of T cells toward antigens presented by DCs, thereby boosting the activation of cytotoxic T lymphocytes. Secretion of cytokines associated with DC maturation was detected. Predictably, consistent with the results of DC maturation and MHC‐II expression, the 2DG@FS‐Nb plus 1064 nm laser irradiation‐treated group presented the highest levels of TNF‐α and IL‐6 (Figure 3j,k). These results confirm that the treatment with 2DG@FS‐Nb plus NIR‐II laser irradiation can significantly induce anti‐tumor immune responses through cascaded activation of ICD and DC maturation, thus promoting the proliferation of cytotoxic T lymphocytes.

To explore the influence of 2DG@FS‐Nb‐mediated glycolytic metabolism regulation on macrophage polarization, RAW 264.7 macrophages were co‐cultured with 4T1 cells with various treatments, and phenotypes of macrophages were identified by analyzing the surface stimulating molecules (Figure 3l). As shown in Figure S20 (Supporting Information) and Figure 3m, after incubating macrophages with tumor cells without any treatments (control group), we observed a higher proportion of M2 macrophages, possibly ascribed to the aggressive M2 polarization caused by excess LA in the TME. However, 2DG, 2DG@FS, and 2DG@FS‐Nb plus laser irradiation groups completely reversed the M1/M2 ratio and displayed more populations of M1 macrophages (Figure 3n), indicating the polarization trends of the M2 phenotype into the M1 phenotype. This confirms that obstruction of 2DG@FS‐Nb‐mediated glycolysis can significantly promote the polarization of TAMs from the M2 phenotype to the M1 phenotype, thus remodeling immunosuppressive TME.

### 2.7 NIR‐II FI and NIR‐II PAI In Vitro and In Vivo

**Figure 4:**
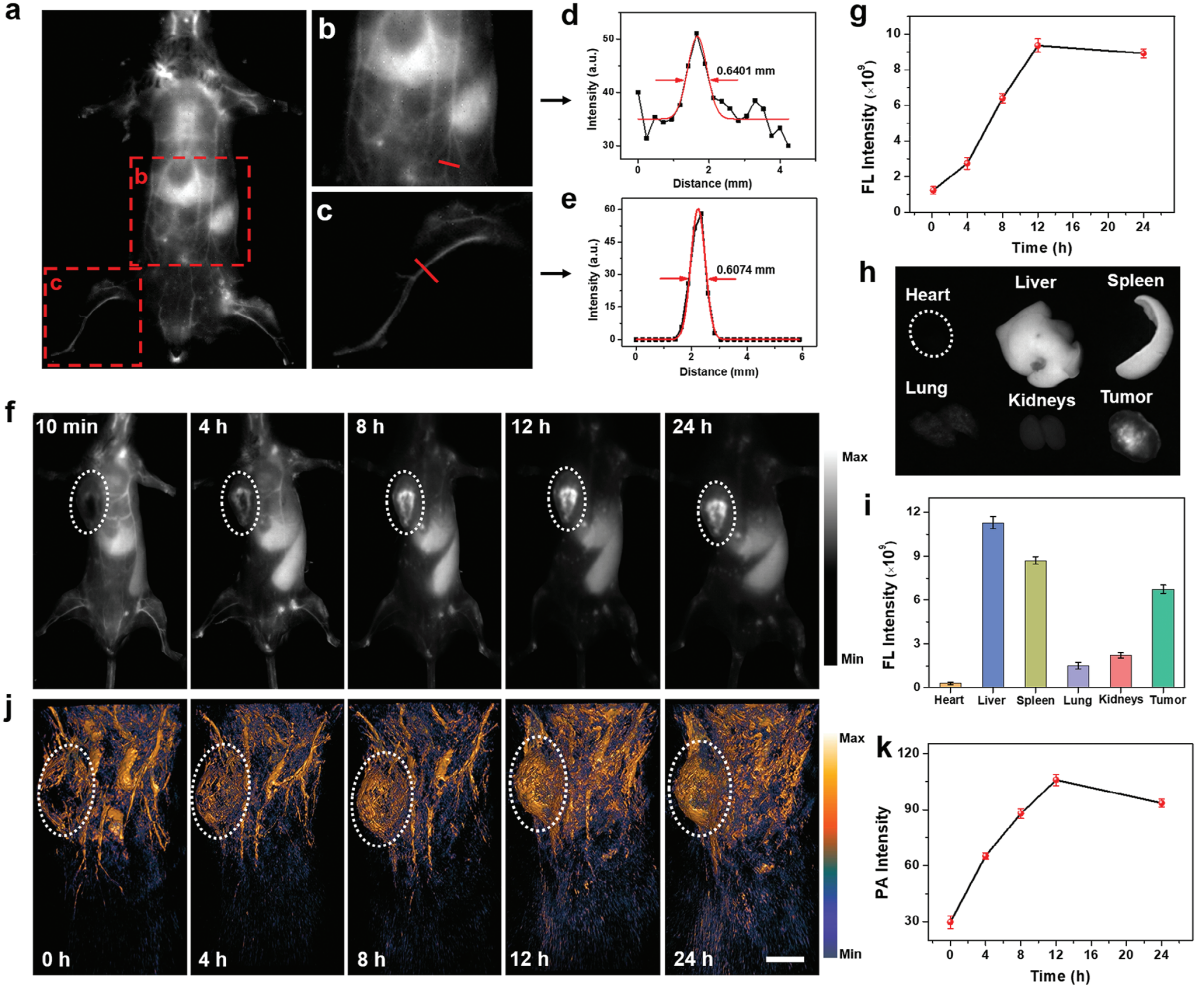
NIR‐II FI/PAI in vivo. a) NIR‐II fluorescence images of the whole mouse body, as well as b) the abdomen and c) hind‐limb vessels 10 min after injection with 2DG@FS‐Nb via the tail vein. d,e) FWHM analysis corresponding to the labeled vessels in (b,c). f) NIR‐II fluorescence images and g) quantitative tumor fluorescence intensity analysis of 4T1 tumor‐bearing mice after intravenous injection with 2DG@FS‐Nb. h) NIR‐II fluorescence images and i) quantitative intensity analysis of the tumor and major organs at 24 h post‐injection. j) NIR‐II PA images of the whole mouse body and k) PA intensity analysis of tumors over time. Scale bar: 5 mm.

To evaluate Nb‐mediated active tumor targeting ability, 4T1 cells were incubated with 2DG@FS and 2DG@FS‐Nb, and NIR‐II FI of cells was detected. A higher NIR‐II fluorescence signal was observed in the cells treated with 2DG@FS‐Nb, with a 1.96‐fold enhancement compared to 2DG@FS‐treated group (Figure S21, Supporting Information), due to the active binding of Nb to PD‐L1 expressed on tumor cells. Meanwhile, 4T1 cells treated with 2DG@FS‐Nb showed the concentration‐dependent NIR‐II fluorescence images (Figure S22, Supporting Information), indicating the excellent intracellular uptake capability of 2DG@FS‐Nb for visualization of deep‐seated tumor cells via NIR‐II FI. To explore the feasibility of 2DG@FS‐Nb for NIR‐II FI in vivo, NIR‐II FI of the mouse vasculature was performed after 10 min via tail vein injection. The systemic blood vessels of the mouse, especially the abdominal and hindlimb vasculatures, were clearly visualized with a high contrast (**Figure** **4**a–c). In the labeled vessels, the full widths at half‐maximum (FWHM) of the cross‐sectional intensity profile were measured to be ≈0.640 and 0.607 mm, respectively, indicating high imaging resolution and SBR (Figure 4d,e). To provide precise guidance for synergistic anti‐tumor immunotherapy, the xenograft 4T1 tumor models were established for further investigating NIR‐II FI capability of 2DG@FS‐Nb. After intravenous injection, the NIR‐II fluorescence brightness of tumor areas increased in a time‐dependent manner and peaked at 12 h post‐injection (Figure 4f), with an ≈7.49‐fold enhancement compared to that at 10 min post‐injection (Figure 4g). At 24 h post‐injection, the liver, spleen, and tumor area exhibited higher NIR‐II fluorescence signals compared to other organs (Figure 4h,i), verifying the preferential accumulation of 2DG@FS‐Nb in these organs and providing effective guidance for pharmacokinetic assessment. In addition, NIR‐II PAI capability of tumors mediated by 2DG@FS‐Nb was also investigated. As shown in Figure 4j,k, the microscopic blood vessels of the tumor were clearly identified at 4 h post‐injection, and the abundance of tumor vessels was gradually improved through enhanced NIR‐II PA signals, and peaked at 12 h post‐injection, implying the maximum accumulation of 2DG@FS‐Nb in the tumor area. These results confirm the excellent NIR‐II FI/PAI performance of 2DG@FS‐Nb for deep‐seated tumor tissues owing to the powerful tissue penetration ability of NIR‐II light accompanied by suppressed photon scattering.

### 2.8 In Vivo Synergistic Anti‐Tumor Immunotherapy

**Figure 5:**
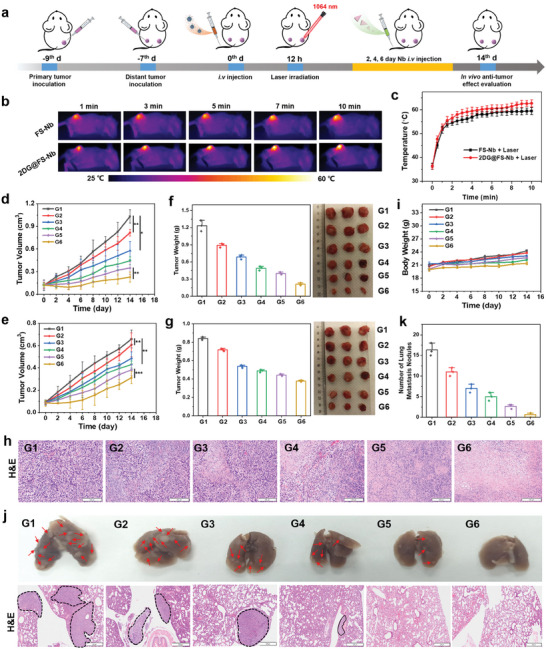
In vivo synergistic anti‐tumor therapy. a) The therapeutic process of 2DG@FS‐Nb‐mediated synergistic therapy against bilateral tumors. b) Infrared thermal images and c) temperature variation of primary tumors treated with FS‐Nb and 2DG@FS‐Nb under 1064 nm laser irradiation (1 W cm^−2^). Size changes of d) primary and e) distant tumors. Tumor weight of f) primary and g) distant tumors after 14 days of treatment. h) H&E staining images of primary tumor sections after 14 days. Scale bar: 200 µm. i) Body weight of mice over 14 days. j) Representative photographs and H&E staining images of lung metastasis (Scale bar: 500 µm), as well as k) statistical analysis of lung metastatic nodules after 14 days of treatment. G1: PBS; G2: Nb; G3: FS‐Nb; G4: 2DG@FS‐Nb; G5: FS‐Nb + Laser; G6: 2DG@FS‐Nb + Laser.

To evaluate the anti‐tumor therapeutic effect and immune response of 2DG@FS‐Nb, a bilateral syngeneic 4T1 tumor‐bearing BALB/c mouse model (primary and distant) was established. The 4T1 tumor‐bearing mice were divided into six groups and underwent different treatments. The treatment process is depicted in **Figure** **5**a. At 12 h post‐injection, 1064 nm laser irradiation was implemented on the primary tumors of FS‐Nb and 2DG@FS‐Nb injected groups (1 W cm^−2^, 10 min), and infrared thermal images of the tumor area were obtained (Figure 5b). The temperature of the tumor can quickly rise to 50 °C within 1 min for effective tumor ablation (Figure 5c). Volume changes in primary and distant tumors were continuously monitored after various treatments. As shown in Figure 5d,e, compared to the PBS group, visible tumor growth inhibition of primary and distant tumors was observed in the groups treated with Nb and FS‐Nb, owing to the combination therapeutic efficiency of ICB and ferroptosis. Notably, laser irradiation can significantly enhance tumor growth inhibition because of the NIR‐II PTT‐induced synergistic immune response. Importantly, compared to FS‐Nb plus laser irradiation, 2DG@FS‐Nb plus laser irradiation displayed higher growth inhibition toward primary and distant tumors, with an inhibition rate of ≈77.92% and 52.62%, respectively, ascribed to 2DG‐mediated metabolism disturbance of glycolysis and subsequent modulation of the immunosuppressive TME. After 14 days of treatment, various treatment groups exhibited significantly different tumor size by tumor photographs (Figure 5f,g). 2DG@FS‐Nb plus laser irradiation‐treated group showed much lower tumor weights (0.23 and 0.36 g for primary and distant tumors, respectively), compared to PBS‐treated group. H&E staining images revealed the most severe cell injury and necrosis of tumor tissues in the group treated with 2DG@FS‐Nb plus laser irradiation (Figure 5h; Figure S23, Supporting Information). After various treatments, all groups remained fundamentally stable body weight within 14 days (Figure 5i). Moreover, no observable damage was found in the major organs (heart, liver, spleen, and kidney) through pathological analysis of H&E staining (Figure S24, Supporting Information). In addition, 2DG@FS‐Nb exhibited low hemolysis rate at various concentrations (Figure S25, Supporting Information). These results verify good biocompatibility and biosafety of 2DG@FS‐Nb.

The anti‐metastatic effect was evaluated by investigating lung metastasis after 14 days of treatment. More lung metastasis nodules were observed in the PBS‐ and Nb‐treated groups (Figure 5j,k), indicating a weak immune activation. However, tumor metastasis was effectively suppressed in the other treatment groups. Notably, 2DG@FS‐Nb plus laser irradiation group exhibited negligible metastatic nodules, as evidenced by the H&E staining images of the lungs. These results confirm the excellent anti‐tumor therapeutic effect of 2DG@FS‐Nb toward primary and distant tumors with the significant anti‐metastatic capability under 1064 nm laser irradiation.

### 2.9 Therapeutic Mechanisms of 2DG@FS‐Nb for Amplified Immunotherapy

**Figure 6:**
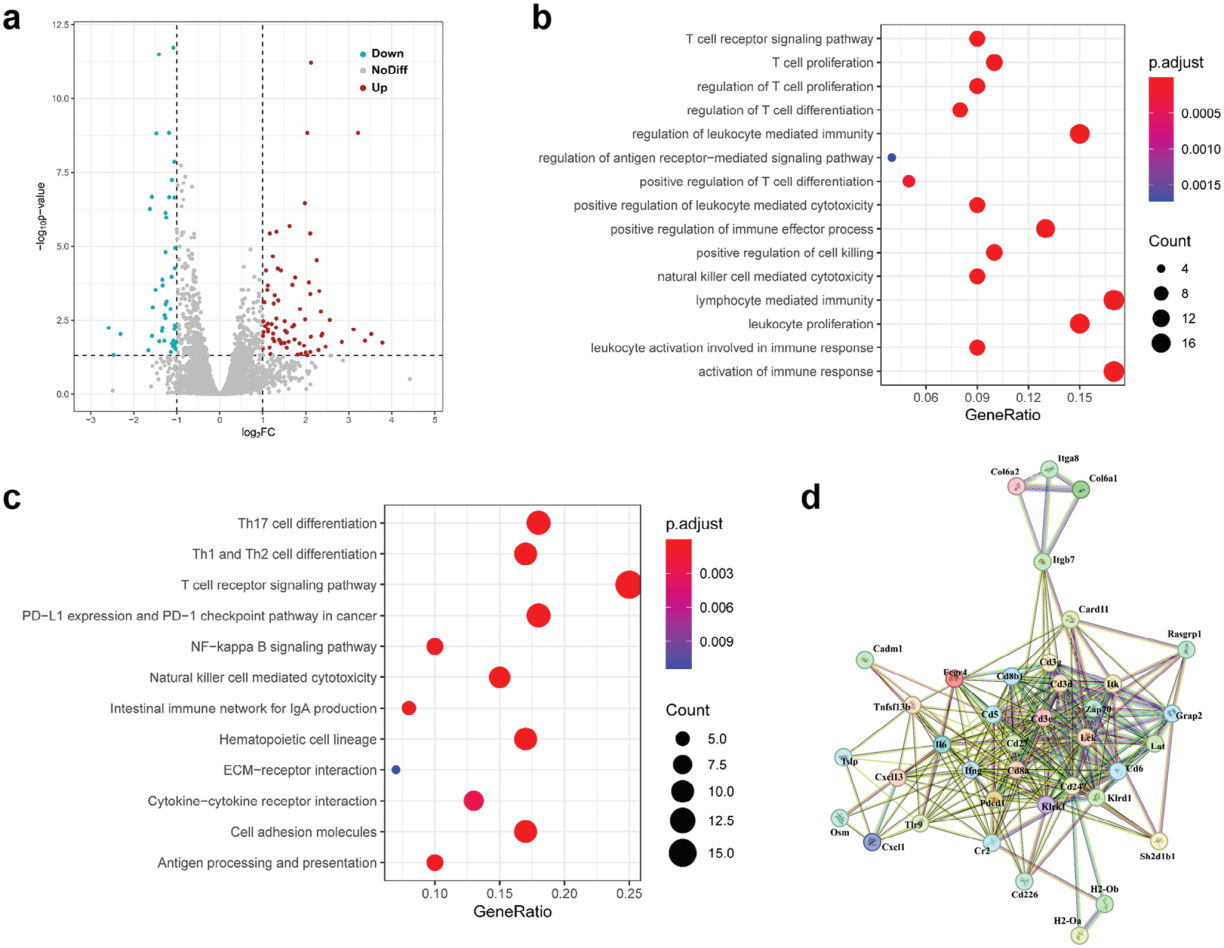
mRNA sequencing analysis between PBS group and 2DG@FS‐Nb + Laser group. a) Volcano map of DEGs, b) GO enrichment analysis, c) KEGG pathway enrichment analysis and d) PPI analysis.

Transcriptomic sequencing was implemented to investigate the potential mechanism of 2DG@FS‐Nb‐mediated amplified immunotherapy. Remarkably differentially expressed genes (DEGs) associated with immune activation and immune regulation were screened between PBS group and 2DG@FS‐Nb + Laser group (**Figure** **6**a; Figure S26, Supporting Information). Gene Ontology (GO) enrichment analysis revealed that 2DG@FS‐Nb + Laser significantly altered the genes related to immune activation and the genes associated with the proliferation, differentiation and regulation of T cells (Figure 6b). This result suggested that 2DG@FS‐Nb can activate the anti‐tumor immune response and promote the production and proliferation of cytotoxic T cells, which may be attributed to the enhanced antigen presentation and T lymphocyte recruitment triggered by ICD. In addition, DEGs related to leukocyte activation and leukocyte mediated cytotoxicity implied that the enhanced immune response may be attributed to the fact that 2DG‐mediated lactate metabolism regulation further reversed macrophage polarization, thereby amplifying the efficiency of immunotherapy. Kyoto Encyclopedia of Genes and Genomes (KEGG) pathway enrichment analysis confirmed the significant changes of the T cell receptor signaling pathway and PD‐1/PD‐L1 checkpoint pathway, accompanied by the differentiation of helper T cells (Th17, Th1 and Th2 cells) (Figure 6c), which may be ascribed to that aPD‐L1 nanobody modified on the surface of 2DG@FS‐Nb promoted the intratumoral infiltration of T cells by ICB. Protein‐protein interaction (PPI) network analysis indicated that key proteins, such as CD3G, CD3D, CD8a, PDCD1, and IFNG, were closely related to T cell proliferation and cytokine secretion (Figure 6d). These results confirm that the Nb‐engineered nanoadjuvants afford amplified efficiency of anti‐tumor immunotherapy under NIR‐II laser irradiation by remodeling immunosuppressive TME in combination with Nb‐mediated ICB.

### 2.10 In Vivo Immunogenic Activation

**Figure 7:**
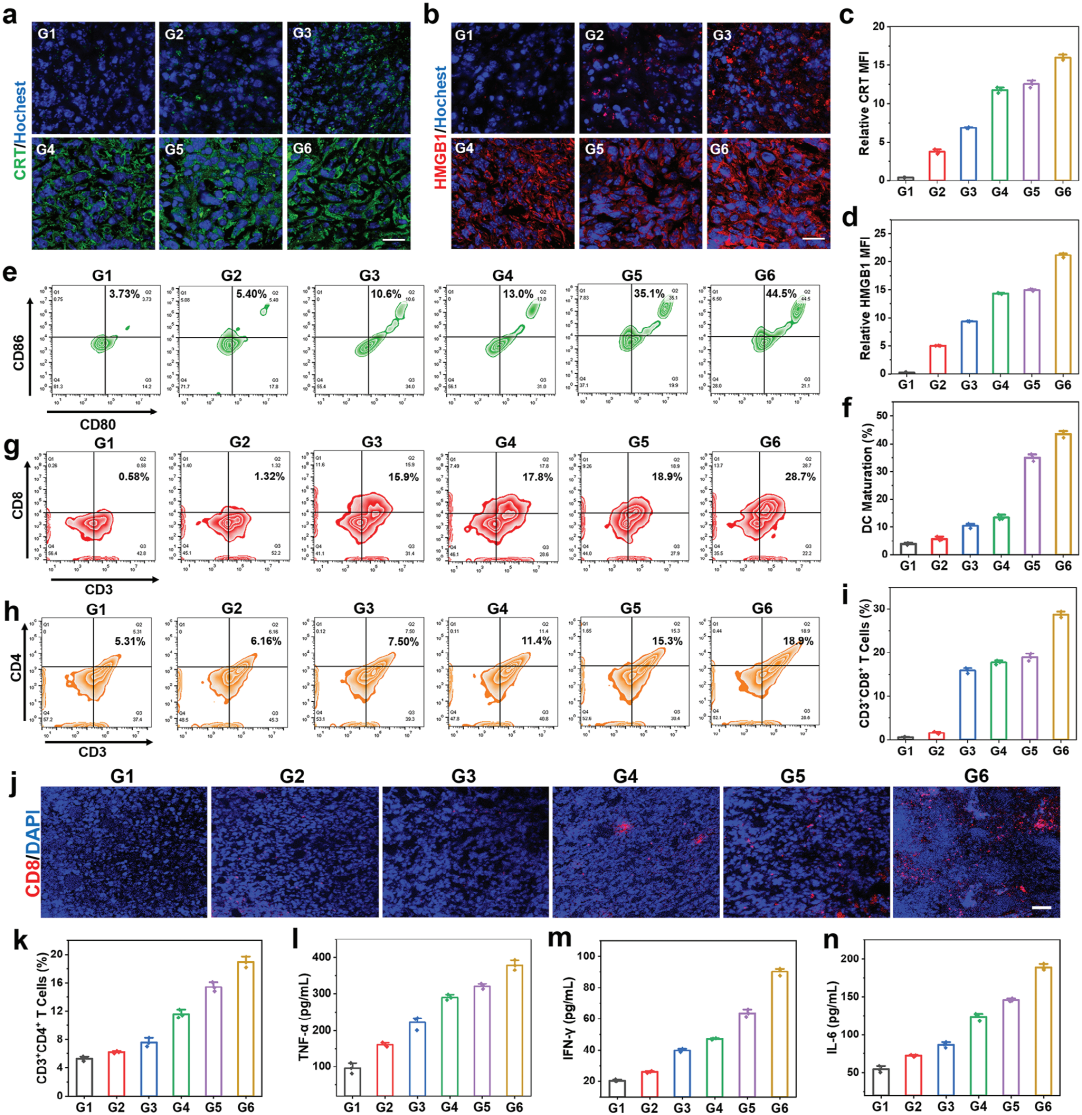
In vivo immunogenic activation. a,b) Immunofluorescence staining images and c,d) quantitative fluorescence intensity analysis of CRT and HMGB1 in primary tumors after 7 days of treatments. Scale bar: 20 µm. e) Flow cytometric plots and f) statistical analysis of DC maturation in primary tumor‐draining lymph nodes. g,h) Flow cytometry assay and i,k) corresponding statistical data of CD3^+^CD8^+^ and CD3^+^CD4^+^ T cells in primary tumor tissues after 7 days of treatment. j) Immunofluorescence staining images of CD8^+^ T cells in primary tumor tissues. Scale bar: 50 µm. Levels of l) TNF‐*α*, m) IFN‐*γ*, and n) IL‐6 in the serum of mice using ELISA after 14 days of treatment. G1: PBS; G2: Nb; G3: FS‐Nb; G4: 2DG@FS‐Nb; G5: FS‐Nb + Laser; G6: 2DG@FS‐Nb + Laser.

To explore the mechanism of anti‐tumor immune response induced by 2DG@FS‐Nb, ICD induced by synergistic therapy was first investigated by collecting primary tumors after 7 days of treatment. As shown in **Figure** **7**a,b, intense fluorescence signals of CRT and HMGB1 were detected in the 2DG@FS‐Nb, FS‐Nb + Laser, and 2DG@FS‐Nb + Laser treated groups, indicating efficient release of CRT and HMGB1 upon various treatment modes. The group treated with 2DG@FS‐Nb + Laser exhibited the highest fluorescence signal of CRT and HMGB1, with ≈1.28‐ and 1.42‐fold enhancement of fluorescence intensity (Figure 7c,d), respectively, compared to the FS‐Nb + Laser treated group. The release of these DAMPs can promote the uptake and presentation of tumor‐associated antigens (TAAs) by DCs, thereby activating cytotoxic T lymphocytes. The maturation of DCs based on ICD in tumor‐draining lymph nodes was investigated after various treatments (Figure S27a, Supporting Information). Obvious increase in the proportion of mature DCs (CD11c^+^CD80^+^CD86^+^) were observed in the 2DG@FS‐Nb‐treated group (Figure 7e), owing to the combined therapeutic effects of ferroptosis and glycolysis inhibition. The 2DG@FS‐Nb + Laser‐treated group exhibited the highest population of mature DCs, increasing by ≈11.93‐ and 1.27‐fold compared to the PBS and FS‐Nb + Laser groups (Figure 7f), respectively. This verified that 2DG@FS‐Nb can provoke effective DC maturation under the synergistic therapeutic effect of NIR‐II PTT‐ferroptosis and glycolysis metabolism disorder. The infiltration of CD8^+^ and CD4^+^ T cells in primary and distant tumors was further evaluated after various treatments (Figure S27b, Supporting Information). As indicated in Figure 7g,h, higher intratumoral infiltration populations of CD8^+^ T and CD4^+^ T cells were detected in primary tumors treated with 2DG@FS‐Nb and FS‐Nb + Laser compared to the PBS‐treated group. Notably, the proportion of CD8^+^ T and CD4^+^ T cells in the 2DG@FS‐Nb + Laser treated group was ≈1.52‐ and 1.24‐fold higher than that in the group treated with FS‐Nb + Laser (Figure 7i,k), possibly ascribed to the augmented immune response through 2DG‐mediated remodeling of immunosuppressive TME, thereby promoting the proliferation and infiltration of CTLs. Immunofluorescence staining images revealed the highest infiltration of CD8^+^ T cells in the group treated with 2DG@FS‐Nb + Laser (Figure 7j). The infiltration of CD8^+^ T cells and CD4^+^ T cells in distant tumors exhibited similar results to those in primary tumors (Figure S28, Supporting Information). Furthermore, the secretion of immune‐related cytokines accompanied by CTL infiltration was measured in serum collected after various treatments. The 2DG@FS‐Nb + Laser treated group presented the highest levels of TNF‐α, IFN‐γ, and IL‐6 (Figure 7l–n), implying the immune‐strengthening effect of 2DG@FS‐Nb plus NIR‐II laser irradiation. These results demonstrate that 2DG@FS‐Nb can evoke augmented anti‐tumor immune responses through NIR‐II photothermal amplified ferroptosis with glycolysis inhibition‐mediated immune remodeling.

### 2.11 In Vivo Immune Remodeling

**Figure 8:**
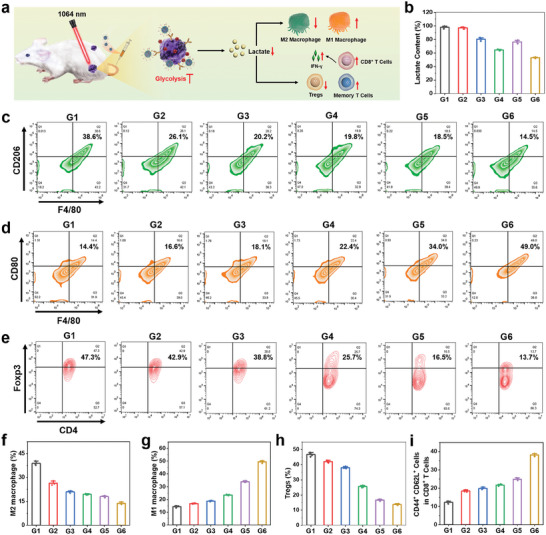
In vivo immune remodeling. a) Schematic illustration of 2DG@FS‐Nb‐mediated amplified immunotherapy by regulating LA metabolism. b) LA content in tumor tissues after 7 days of different treatments. c,d) Flow cytometry analysis and f,g) statistical results of M1 macrophages (F4/80^+^CD80^+^) and M2 macrophages (F4/80^+^CD206^+^) in tumor tissues. e) Flow cytometry detection and h) statistical results of tumor‐infiltrating Tregs (CD3^+^CD4^+^Foxp3^+^). i) Quantification of central memory T cells (CD3^+^CD8^+^CD44^+^CD62L^+^) in various treatment groups. G1: PBS; G2: Nb; G3: FS‐Nb; G4: 2DG@FS‐Nb; G5: FS‐Nb + Laser; G6: 2DG@FS‐Nb + Laser.

The immune remodeling ability of 2DG@FS‐Nb was evaluated after 7 days of treatment (**Figure** **8**a). As an important marker in immunosuppressed TME, the content of LA in primary tumor tissues was first detected. The groups treated with 2DG@FS‐Nb exhibited relatively lower LA levels with or without 1064 nm laser irradiation compared to the other treatment groups (Figure 8b), suggesting that the introduction of the glycolysis inhibitor 2DG can significantly hinder the production of LA in tumor tissues, thus reshaping immunosuppressive TME and enhancing anti‐tumor immune responses. The proportion of M2 macrophages in the FS‐Nb + Laser group drastically decreased from 38.6% in the PBS group to 18.5% (Figure S29, Supporting Information and Figure 8c), due to ICD induced by NIR‐II PTT amplified ferroptosis, followed by the activation of CTLs to secrete IFN‐γ, which can promote the polarization of M2 macrophages into M1 macrophages. Moreover, the 2DG@FS‐Nb + Laser group exhibited the lowest population of M2 macrophages, which decreased by ≈2.66‐fold compared to the PBS group (Figure 8f). This might be ascribed to the reduced LA generation by 2DG‐mediated glycolysis inhibition, thus attenuating M2 macrophage polarization by relieving immunosuppression in the TME. Accordingly, obviously improved populations of M1 macrophages were detected in the treatment groups (Figure 8d). Notably, an ≈3.40‐fold increase in the proportion of M1 macrophages was observed in the 2DG@FS‐Nb + Laser group compared to the PBS group (Figure 8g). The upregulation of the M1/M2 ratio indicates the alleviation of the immunosuppressive TME in tumor tissues, contributing to the inhibition of tumor growth and metastasis. Additionally, regulatory T cells (Tregs) with negative immune regulation were detected. 2DG@FS‐Nb treatment induced the least Treg (CD4^+^Foxp3^+^) generation under 1064 nm laser irradiation, with a 3.45‐fold decrease compared to that in the PBS group (Figure 8e,h), as evidenced by the negligible immunofluorescence of Tregs in tumor tissues (Figure S30, Supporting Information). To investigate the potential of synergistic therapy in promoting long‐term immunological memory, after 14 days of treatment, spleens from all groups of mice were collected for evaluation of central memory T cells (T_cm_). As shown in Figure S31 (Supporting Information) and Figure 8i, the groups treated with FS‐Nb and 2DG@FS‐Nb with or without NIR‐II laser irradiation exhibited remarkably higher levels of T_cm_ (CD44^+^CD62L^+^) than the other groups, demonstrating the ability of 2DG@FS‐Nb to activate lasting anti‐tumor immune response under NIR‐II laser irradiation. These results confirm that 2DG@FS‐Nb can remodel and reverse the immunosuppressive TME and restore CD8^+^ T cell activity through glycolytic metabolism regulation, thus acquiring amplified anti‐tumor immune output.

## 3 Conclusion

In this study, a novel immunomodulatory NIR‐II nanoadjuvant (2DG@FS‐Nb) with TME reprogramming capability was prepared through metallic iron ion‐driven coordination self‐assembly of NIR‐II molecules, followed by loaded with glycolysis inhibitors and modified with Nb. After Nb‐mediated positive tumor targeting and ICB, followed by TME‐triggered rapid cargo release, 2DG@FS‐Nb enabled NIR‐II FI/PAI‐guided NIR‐II PTT amplified ferroptosis, provoking robust ICD and immune responses. More importantly, 2DG@FS‐Nb modulated and remodeled the immunosuppressive TME through LA efflux inhibition, thereby synergistically amplifying the efficiency of immunotherapy. This study provided a new strategy to augment immune output through the integration of NIR‐II phototheranostics and tumor metabolic reprogramming in combination with ICB.

## Conflict of Interest

The authors declare no conflict of interest.

## Supporting information

Supporting Information

## Data Availability

The data that support the findings of this study are available from the corresponding author upon reasonable request.
