# Supplementary material for: Metal‐Coordinated NIR‐II Nanoadjuvants with Nanobody Conjugation for Potentiating Immunotherapy by Tumor Metabolism Reprogramming
Source: Adv Sci (Weinh). 2024 Jul 7;11(34):2404886. doi: 10.1002/advs.202404886 (PMC11425641; doi:10.1002/advs.202404886)
Supplement: Supplementary file 1 — Supporting Information [file ADVS-11-2404886-s001.docx]

**Metal-coordinated NIR-II** **Nanoadjuvants with Nanobody Conjugation for** **Potentiating Immunotherapy by Tumor Metabolism Reprogramming**

*Yeneng Dai, Ziang Guo, Dongliang Leng, Guanda Jiao, Kai Chen, Mingxuan Fu, Yang Liu, Qingming Shen, Qi Wang,* Lipeng Zhu,* Qi Zhao**

Cancer Centre, Institute of Translational Medicine, Faculty of Health Sciences, University of Macau, Macau SAR 999078, China.

E-mail: qizhao@um.edu.mo

State Key Laboratory of Organic Electronics and Information Displays & Jiangsu Key Laboratory for Biosensors, Institute of Advanced Materials (IAM), Nanjing University of Posts & Telecommunications, Nanjing 210023, China.

1. mail: iamqwang@njupt.edu.cn

MoE Frontiers Science Center for Precision Oncology, University of Macau, Taipa, Macau SAR 999078, China.

School of Life Sciences, Central South University, Changsha 410013, China.

E-mail: zhuleaper@csu.edu.cn

**Experimental section**

**Materials**

FeCl_3_∙6H_2_O, trifluoroacetic acid (TFA), *N*,*N*-Dimethylformamide (DMF), Rhodamine B (RhB), 2-Deoxy-D-glucose (2DG), 5,5'-dithiobis-2-(nitrobenzoic acid) (DTNB) and 3,3',5,5' -tetramethylbenzidine (TMB) were purchased from Aladdin Reagent Co., Ltd. (Shanghai, China). 1,2-distearoyl-*sn*-glycero-3-phosphoethanolamine-N-[carboxy(polyethylene glycol)-5000] (DSPE-PEG_5000_-COOH) was purchased from Shanghai Ponsure Biotech, Inc. Lactic Acid Assay Kit was provided by Nanjing Jiancheng Bioengineering Institute (China). ATP Assay Kit was bought from Beyotime Biotechnology. 2′, 7′-dichlorodihydrofluorescein diacetate (DCFH-DA), Cell Counting Kit-8 (CCK-8), Annexin V-FITC/PI Kit and JC-1 probe were obtained from Yeasen Biotechnology CO., Ltd. (Shanghai, China). FITC-CD11c, PE-CD80, Percp-CD86, PE-CD3, Percp-CD8a, FITC-CD4, FITC-CD3, Percp-CD4, PE-Foxp3, Percp-CD8a, Alexa-Fluor@700 CD44 and APC/Cyanine 7 CD62L were purchased from BioLegend.

**Characterization**

The structure of the NIR-II molecule was determined by ^1^H NMR spectra using the Bruker Ultra Shield Plus NMR instrument (400 MHz). TEM images were acquired using a HT7700 transmission electron microscope (100 KV). The size distribution and zeta potential were measured by Nano-ZS ZEN3600 (Malvern). Shimadzu UV-3600 spectrophotometer and NIR-II spectrophotometer (Fluorolog 3, Horiba) were used to detect UV-vis absorption spectra and fluorescence emission spectra, respectively. NIR-II Fluorescence imaging was performed and analyzed by AniView Phoenix Full Spectrum Animal *In Vivo* Imaging System (Guangzhou Biolight Biotechnology Co., Ltd.). NIR-II photoacoustic imaging was obtained and analyzed using an *in vivo* 3D optoacoustic imaging system (TomoWave Laboratories, Inc.).

**Preparation of aPD-L1 nanobody (Nb)**

The structure and sequence of aPD-L1 nanobody are depicted in Scheme S1 and were prepared according to previously reported literature.^[1]^ Briefly, an OmpA signal peptide was added to the N-terminal of the anti-mouse PD-L1 VHH, while a Flag tag and a hexahistidine (His6) tag were added to the C-terminal. Modified VHH was inserted into the pComb3X vector by molecular cloning. The plasmid was transformed into *Escherichia coli* HB2151 containing 0.5 mM IPTG for further culture overnight at 30 °C at 220 rpm. After centrifugation at 4000 ×*g* for 10 min, the bacteria were lysed with polymyxin B for 30 min at 30 °C. The bacterial lysate was centrifuged at 8000 ×*g* for 10 min and loaded over Ni-NTA according to the manufacturer’s instructions. The resin was washed with buffer (10 mM phosphate buffered saline [PBS] [pH 7.2~7.4], 300 mM NaCl, and 18 mM imidazole) and then eluted with elution buffer (10 mM PBS [pH 7.2~7.4], 300 mM NaCl, and 200 mM imidazole). After purification, the protein was buffered in PBS through ultrafiltration (MW: 3 KDa).

**AA sequence of anti-mouse PD-L1 VHH:**

QVQLVETGGGLVQPGGSLRLSCTASGFTFSMHAMTWYRQAPGKQRELVAVITSHGDRANYTDSVRGRFTISRDNTKNMVYLQMNSLKPEDTAVYYCNVPRYDSWGQGTQVTVSSGQAGQHHHHHHGAYPYDVPDYAS

**Synthesis of the NIR-II molecule Se-TC**

The NIR-II molecule Se-TC was synthesized via the synthetic route shown in Scheme S1. Compound 3 was synthesized from our previously reported work.^[2]^ To obtain carboxyl-grafted NIR-II molecules, compound 3 (40 mg) was dissolved in 15 mL of dichloromethane before adding 4 mL of trifluoroacetic acid (TFA). After stirring for 24 h at room temperature, the solvent was removed by rotary evaporation and a black solid was obtained after further extraction and washing.

**Preparation of 2DG@FS**

To prepare 2DG@FS, 1.5 mL FeCl_3_∙6H_2_O (2 mg/mL, DMF) and 3 mL Se-TC (1 mg/mL, DMF) were mixed in a round bottom flask and stirred for 6 h at 70 °C. After cooling to room temperature, ultrapure water with 3 to 4 times the volume of DMF was added, followed by standing overnight. The mixed solution was centrifuged (11000 rpm, 15 min) to obtain NIR-II self-assembled NPs of Se-TC using Fe^3+^ as the coordination nodes. To obtain water-soluble NPs, 30 mg of DSPE-PEG_5000_-COOH was added to the DMF solution of the self-assembly for further stirring for 3 h. Finally, water-soluble FS NPs were obtained after overnight dialysis (MWCO: 8000~14000).

To load the glycolysis inhibitor 2DG, 2DG aqueous solution (5 mg/mL) was added to the DMF solution of self-assembled Se-TC NPs, then 30 mg DSPE-PEG_5000_-COOH was added. The mixed solution was stirred for 3 h at room temperature. Finally, the solution was dialyzed overnight (MWCO: 8000~14000) to remove free 2DG and DSPE-PEG_5000_-COOH, ultimately obtaining 2DG@FS NPs.

**Preparation of 2DG@FS-Nb**

To obtain Nb-modified NIR-II nanoadjuvants, 2DG@FS NPs were dissolved in a PBS solution containing EDC (10 mg) and NHS (10 mg), and the mixed solution was stirred for 1 h to activate the carboxyl groups on the surface of the NPs. Next, Nb (1 mg/mL) was added dropwise to the NP solution and further shaken overnight at room temperature. The product was washed three times with water to remove the unbound antibody by ultrafiltration (MW: 50 KDa), obtaining 2DG@FS-Nb. Next, to obtain RhB-doped 2DG@FS-Nb, during the preparation of 2DG@FS, RhB (10 μL, 1 mg/mL) was added to the DMF solution of self-assembled Se-TC NPs together with 2DG to obtain 2DG@FS-Nb with RhB doping.

**Drug release detection**

2DG@FS-Nb was dissolved in PBS with different pH values (pH 7.4 and pH 5.5). At different time points, the mixed solution was centrifuged by ultrafiltration (MW: 3000), and the filtrate was collected and assayed for determination of released 2DG by UV-vis absorption spectrum or HPLC according to previously reported literature.^[3]^

**NIR-II photothermal performance test**

To investigate NIR-II photothermal performance of 2DG@FS-Nb, 2DG@FS-Nb aqueous solutions containing different Se-TC concentrations (25, 50, 100, 150, 200 μg/mL) were exposed to 1064 nm laser and continuously irradiated for 5 min (1 W/cm^2^). The temperature of the solution was monitored using an infrared thermal imaging camera. To investigate the influence of the laser power density on photothermal performance, 1064 nm laser with different power densities (0.5, 1.0, 1.5 W/cm^2^) was irradiated on 2DG@FS-Nb solution (100 μg/mL) and temperature changes were recorded. In order to explore the photostability, 2DG@FS-Nb aqueous solution was irradiated by 1064 nm laser for 5 min (1 W/cm^2^). Then, the laser was turned off and the solution was naturally cooled to room temperature. During five repeated laser on/off cycles, the temperature of the solution was monitored and recorded. The photothermal conversion efficiency (*η*) was further calculated through a heating/cooling cycle according to the following equation.

*η =*

*hS (T_Max_-T_Surr_)-Q_Dis_*

*I (1-10^-A^)*

(1)

***In vitro* detection of hydroxyl radical (∙OH) production**

The ∙OH generated by 2DG@FS-Nb catalyzing H_2_O_2_ was detected using 3,3′,5,5′-tetramethyl-benzidine (TMB) as the capture reagent. Briefly, 100 μL 2DG@FS-Nb (300 μg/mL) was added to H_2_O_2_ solution containing different concentrations, and the entire volume of the solution was 1 mL, followed immediately by the addition of 15 μL TMB solution (2 mg/mL). The absorption of the mixed solution was detected using UV-vis absorption spectrophotometer. In order to explore the effect of NIR-II laser irradiation on ∙OH production, the mixed solution of 2DG@FS-Nb and H_2_O_2_ containing TMB was irradiated with or without 1064 nm laser for 10 min (1 W/cm^2^), and the absorption of the solution was further detected.

**Cellular uptake and targeting analysis**

In order to investigate the targeting ability of 2DG@FS-Nb towards tumor cells due to the modification of aPD-L1 nanobody, 2DG@FS (80 μg/mL) and 2DG@FS-Nb (80 μg/mL) with RhB doping were added to 4T1 cells, respectively. After 12 h of incubation, 4T1 cells were washed with fresh PBS, intracellular RhB fluorescence was detected and analyzed by flow cytometry. In addition, about 3000 4T1 cells were seeded on a sphere-ultra-low adsorption surface 96-well plate (Thermo Fisher Scientific, USA), followed by centrifugation at 300 *×g* for 5 min. After about 7 days of incubation, the formed 3D multicellular spheroids were processed according to the protocol described above, and cells were observed under the confocal laser scanning microscope (CLSM).

**Extracellular lactate and intracellular ATP content measurement**

After seeding in 12-well plates for 24 h, 4T1 cells were incubated with FS, 2DG@FS, and 2DG@FS-Nb (2DG: 4 mM) for 24 h. Cell supernatants were collected for the determination of LA content using the Lactic Acid Assay Kit. All cells were lysed to determine the intracellular ATP levels using the ATP Assay Kit according to the manufacturer’s instructions. Furthermore, to investigate the effect of 2DG on LA expression, 4T1 cells were incubated with 2DG@FS-Nb at different 2DG concentrations. After incubation for 24 h, the cell supernatant samples were collected for LA quantification.

**Evaluation of intracellular GSH depletion**

To investigate intracellular GSH depletion after various treatments, 4T1 cells were seeded in 6-well plates and cultured for 24 h. The cells were then incubated with FS-Nb and 2DG@FS-Nb (80 μg/mL) for 12 h. Next, 4T1 cells treated with 2DG@FS-Nb were exposed to 1064 nm laser irradiation for 5 min (1 W/cm^2^). All cells were washed with PBS three times after removing the culture medium, before lysing and centrifuging at 6000 rpm for 5 min. The supernatant was collected and mixed with DTNB solution to determine the intracellular GSH content by measuring the absorbance of the solution at 405 nm using a multifunctional microplate reader.

**Intracellular GPX4 expression by western blotting and immunofluorescence staining**

Briefly, 4T1 cells were seeded in 6-well plates, after incubation for 24 h, FS-Nb and 2DG@FS-Nb (80 μg/mL) were added to the cells, respectively for further incubation for 12 h. Next, 2DG@FS-Nb-treated cells were irradiated with 1064 nm laser for 5 min (1 W/cm^2^). Subsequently, the cells were lysed in radioimmunoprecipitation assay (RIPA) lysis buffer and centrifuged at 12,000 ×*g* for 5 min. The supernatant was collected for protein separation using 10% SDS-PAGE. Separated proteins were transferred to a polyvinylidene fluoride (PVDF) membrane, followed by blocked with 5% skim milk powder for 2 h at room temperature. After washed with PBST three times, the membrane was incubated with GPX4 primary antibody (67763-1-Ig, Proteintech) overnight at 4°C. After washed with PBST three times, the membrane was incubated with HRP goat anti-mouse IgG (H+L) (AS003, ABclonal) for 2 h at room temperature. After further washed with PBST, an enhanced chemiluminescence system was used to visualize the protein bands.

Intracellular GPX4 expression was further validated by immunofluorescence staining. After various treatments, 4T1 cells were fixed with 4% paraformaldehyde for 25 min at room temperature, permeabilized with 0.25% Triton X-100 for 30 min, and blocked with 2.5% bovine serum albumin (BSA) for 2 h. After washed with PBS three times, the cells were incubated with GPX4 monoclonal antibody (67763-1-Ig, Proteintech) overnight at 4°C, followed by incubation with fluorescence-labeled secondary antibody (Goat Anti-Mouse IgG H&L (Alexa Fluor® 647)) for 2 h at room temperature. Finally, all groups of cells were observed under CLSM after nuclear staining with DAPI.

**Intracellular reactive oxygen species production and lipid peroxide measurement**

For intracellular ROS detection, 4T1 cells were seeded in confocal dishes and incubated for 24 h. Then, the cells were treated with FS, 2DG@FS and 2DG@FS-Nb (80 μg/mL) with or without 1064 nm laser irradiation (1 W/cm^2^, 5 min), followed by stained with DCFH-DA for 10 min, and then observed under CLSM. For lipid peroxidation detection, cells subjected to various treatments were stained with 5 μM C11-BODIPY^581/591^ for 30 min, and intracellular fluorescence was observed under CLSM.

**Intracellular mitochondrial membrane potential measurement**

To evaluate intracellular mitochondrial membrane potential after various treatments, 4T1 cells were seeded in confocal dishes. After 24 h of incubation, cells were incubated with FS, 2DG@FS, and 2DG@FS-Nb for 12 h. 2DG@FS-Nb treated cells were exposed to 1064 nm laser irradiation for 5 min (1 W/cm^2^). Then, the cells were stained with JC-1 probe for 15 min, and the intracellular fluorescence was observed by CLSM. (JC-1 monomer: Ex/Em=480 nm/530 nm, JC-1 aggregate: Ex/Em=550 nm/590 nm).

**Cytotoxicity evaluation**

4T1 cells were seeded in 96-well plates and cultured for 24 h. Then, cells were treated with different concentrations of FS-Nb, 2DG@FS-Nb, respectively, followed by irradiated by 1064 nm laser (1 W/cm^2^, 5 min). 10 μL CCK-8 was added to all wells for further incubation for 2 h, cytotoxicity resulted from various treatments was evaluated by the absorbance at 450 nm using a microplate reader. Calcein-AM/PI double-staining assay was used to visualize cytotoxicity. 4T1 cells were seeded in confocal dishes and subjected to various treatments. Cells were stained with calcein-AM and PI, and observed under CLSM. Annexin V-FITC/PI apoptosis detection kit was used to analyze cell apoptosis. After various treatments, all groups of 4T1 cells were digested with EDTA-free trypsin and collected by centrifugation. After stained with Annexin V-FITC and PI, cell apoptosis was analyzed by flow cytometry.

***In vitro* ICD effect**

To evaluate the ICD effect induced by 2DG@FS-Nb-mediated synergistic therapy, the expression levels of ICD markers, including CRT and HMGB1, were detected by immunofluorescence staining. Briefly, 4T1 cells were seeded in confocal dishes and incubated for 24 h, then cells were incubated with FS-Nb and 2DG@FS-Nb (100 μg/mL) for 12 h, followed by irradiated with or without a 1064 nm laser for 5 min (1 W/cm^2^). After various treatments, the cells were fixed with 4% paraformaldehyde for 30 min at room temperature, followed by permeabilization with 0.25% Triton X-100 for 30 min. After washed with PBS three times, the cells were blocked with 2.5% BSA for 2 h. Subsequently, the cells were incubated with CRT or HMGB1 primary antibody overnight at 4 °C and then incubated with the corresponding secondary antibody for 2 h: Goat Anti-Mouse IgG H&L (Alexa Fluor® 647) for CRT staining or FITC-labeled Goat Anti-Mouse IgG (H&L) for HMGB1 staining. All groups of cells were observed by CLSM after nuclear staining with DAPI.

***In vitro* bone marrow-derived DC (BMDC) stimulation**

BMDCs were first extracted from the hind limbs of female BALB/c mice and cultured in the lower chamber of the Transwell system. Next, 4T1 cells were seeded in the upper chamber of the Transwell system and treated with FS-Nb and 2DG@FS-Nb (100 μg/mL). After 12 h of incubation, 1064 nm laser irradiation (1 W/cm^2^, 10 min) was implemented on the cells treated with 2DG@FS-Nb. After co-incubation for 24 h, the DCs in the lower chamber were collected by centrifugation and stained with FITC-CD11c, PE-CD80, and Percp-CD86, followed by flow cytometry analysis. The culture medium was collected to determine the content of IL-6 and TNF-α using ELISA. DCs were stained with PE-MHC-II to detect MHC-II expression on the surface of DCs.

***In vitro* macrophage polarization measurement**

RAW 264.7 macrophages were seeded in the lower compartment of the Transwell systems. 4T1 cells were seeded in upper compartment and treated with 2DG (1 mg/mL), FS-Nb and 2DG@FS-Nb with or without 1064 nm laser irradiation (1 W/cm^2^). After 24 h of co-incubation, macrophages were collected and stained with PE-CD80, and FITC-CD206 for further analysis by flow cytometry.

**Animal Models**

BALB/c mice were provided by Animal Research Core of Faculty of Health Sciences, University of Macau. All animal experiments were approved by the Institutional Animal Care and Use Committee of Macau University, and performed in accordance with the approved protocol (UMARE-041-2020) by the University of Macau Animal Ethics Committee.

***In vitro* and *in vivo* NIR-II FI and NIR-II PAI**

NIR-II FI of 2DG@FS-Nb aqueous solutions with different concentrations (50, 100, 200, 300, 400 μg/mL) was detected under 808 nm laser excitation using the AniView Phoenix Full Spectrum Animal *In Vivo* Imaging System (Guangzhou Biolight Biotechnology Co., Ltd.). NIR-II FI of 4T1 cells treated with 2DG@FS and 2DG@FS-Nb with various concentrations was detected for evaluation of Nb-mediated targeting ability. For *in vivo* NIR-II FI, healthy BALB/c mice were depilated and intravenously injected with 2DG@FS-Nb solution (1 mg/mL, 200 μL) via the tail vein, and NIR-II FI of systemic blood vessels of mice was obtained at 10 min postinjection. The full width at half maximum (FWHM) of tagged vessels in NIR-II images of magnified abdomen and hind limb were analyzed by Image J software. 4T1 tumor-bearing BALB/c mice were intravenously injected with PBS solution of 2DG@FS-Nb (1 mg/mL) through the tail vein. At the indicated time points, NIR-II FI of the mice was detected under 808 nm laser excitation, and the fluorescence intensity of the tumor area was analyzed by the analysis software in the imaging system. NIR-II fluorescence images were obtained using an 1175 nm long pass (LP) filter in all experiments. For *in vivo* NIR-II PAI, after intravenous injection with 2DG@FS-Nb solution (1 mg/mL, 200 μL), the whole-body NIR-II photoacoustic images of 4T1 tumor-bearing mice were obtained under 1064 nm laser excitation at different time points. 3D reconstruction software was used to reconstruct and analyze photoacoustic images through signal conversion.

***In vivo* synergistic anti-tumor therapy and anti-metastasis effect evaluation**

Bilateral tumor models were established to evaluate the synergistic anti-tumor and anti-metastatic therapeutic effects of 2DG@FS-Nb *in vivo*. Briefly, 4T1 cells (1 × 10^6^ cells) were subcutaneously implanted into the right flank of mice as primary tumors. After 2 days, 4T1 cells (5 × 10^5^ cells) were simultaneously subcutaneously implanted into the left flank of mice as distant tumors. After 9 days, all 4T1 tumor-bearing mice were randomly divided into six groups, and the primary tumors were subjected to the following treatments: G1: PBS; G2: Nb; G3: FS-Nb; G4: 2DG@FS-Nb; G5: FS-Nb + Laser; G6: 2DG@FS-Nb + Laser. All samples (approximately 1 mg/mL, 200 μL) were intravenously injected into mice via the tail vein, and the aPD-L1 nanobody (Nb) was intravenously injected into mice on days 0, 2, 4, and 6 at a dose of 1.5 mg/kg. At 12 h post-injection, the primary tumors in the FS-Nb- and 2DG@FS-Nb-treated groups were exposed to 1064 nm laser irradiation for 10 min (1 W/cm^2^), and the time-dependent temperature changes of the tumors were monitored using an infrared thermal imaging camera. After various treatments, the volumes of primary and distant tumors, and the body weights of mice in all groups were continuously monitored within 14 days. After 14 days of treatment, the tumors and major organs of the mice were collected for tumor weighing and H&E staining analysis. The lungs of mice were collected and photographed, and the anti-metastatic effect was evaluated through H&E staining and lung metastatic nodule counting.

**The hemolysis test**

The whole blood was obtained from BALB/c mice and centrifuged at 3000 rpm for 10 min at 4 °C. After washed with PBS, the obtained red blood cells (RBC) were diluted into 5% PBS solution. 2DG@FS-Nb solutions with different concentrations were mixed with an equal volume of RBC solution, followed by incubated for 2 h at 37 °C. After centrifugation, the supernatants of all samples were collected to determine hemolysis rate by the absorption at 540 nm.

***In vivo* lactate measurement**

After 7 days of various treatments, primary tumors from all treatment groups were collected and added to PBS, followed by homogenization under an ice-water bath. Then, the homogenate was centrifuged for 10 min at 2500 rpm, and the supernatant was collected for determination of lactate content using Lactic Acid Assay Kit.

***In vivo* immune response activation and immune remodeling**

After 7 days of treatment, primary tumor tissues of mice were collected for ICD effect evaluation by immunofluorescence staining of CRT and HMGB1. For DC maturation assessment, tumor-draining lymph nodes of primary tumors were collected and prepared into single-cell suspensions, followed by stained with FITC-CD11c, PE-CD80 and Percp-CD86 and analyzed by flow cytometry. For cytotoxic T lymphocyte infiltration, single cell suspensions of primary and distant tumor tissues were stained with PE-CD3, Percp-CD8 and FITC-CD4 and analyzed by flow cytometry.

For the regulation and remodeling of the immunosuppressive TME mediated by 2DG@FS-Nb, after 7 days of treatment, primary tumor tissues were prepared into single cell suspensions, the cells were stained with FITC-CD3, Percp-CD4 and PE-Foxp3 for Treg cell analysis. To evaluate macrophage polarization, cells were stained with APC-CD11b, PE-F4/80 and FITC-CD80 for M1 macrophage evaluation, and APC-CD11b, PE-F4/80 and FITC-CD206 for M2 macrophage evaluation. To investigate the immune memory effect of 2DG@FS-Nb, after 14 days of treatment, cell suspensions of spleens were stained with FITC-CD3, Percp-CD8a, Alexa-Fluor@700 CD44 and APC/Cyanine 7 CD62L, and were further analyzed by flow cytometry.

**Statistical analysis**

The experiments were repeated at least three times. All results were obtained as means and standard deviations calculated from the measurements. Data were analyzed via ANOVA (comparing more than two samples), statistical significance was defined as **p* < 0.05, ***p* < 0.01 and ****p* < 0.001.


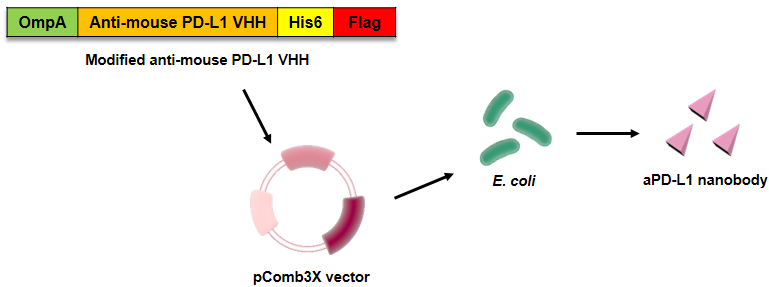


**Scheme S1.** The structure and preparation process of aPD-L1 nanobody (Nb).


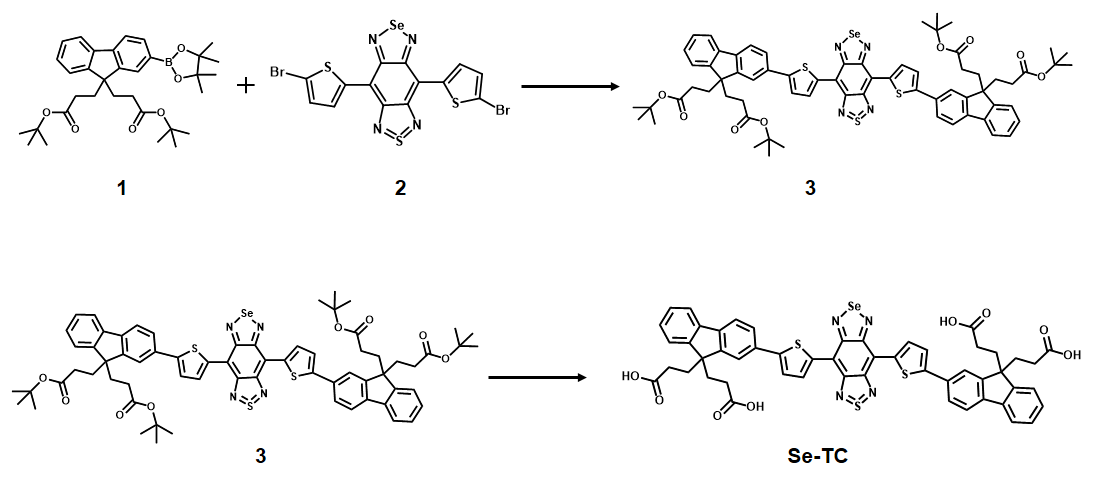


**Scheme S2.** Synthetic route of NIR-II molecule Se-TC.


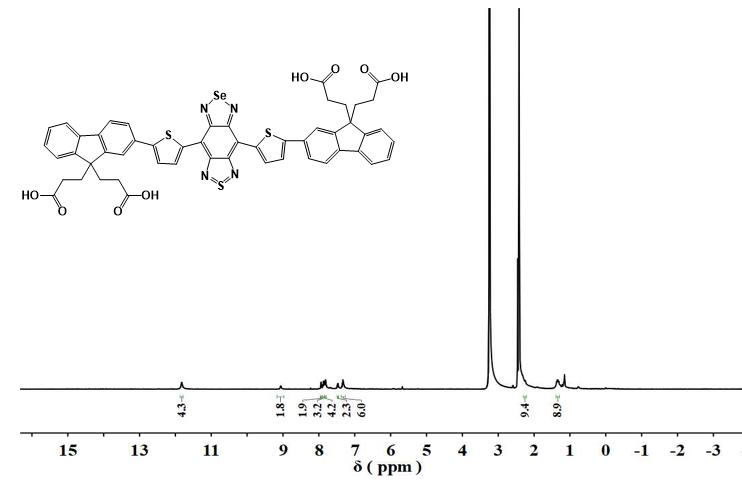


**Figure S1.** ^1^H NMR spectrum (DMSO-*d*_6_) of Se-TC.


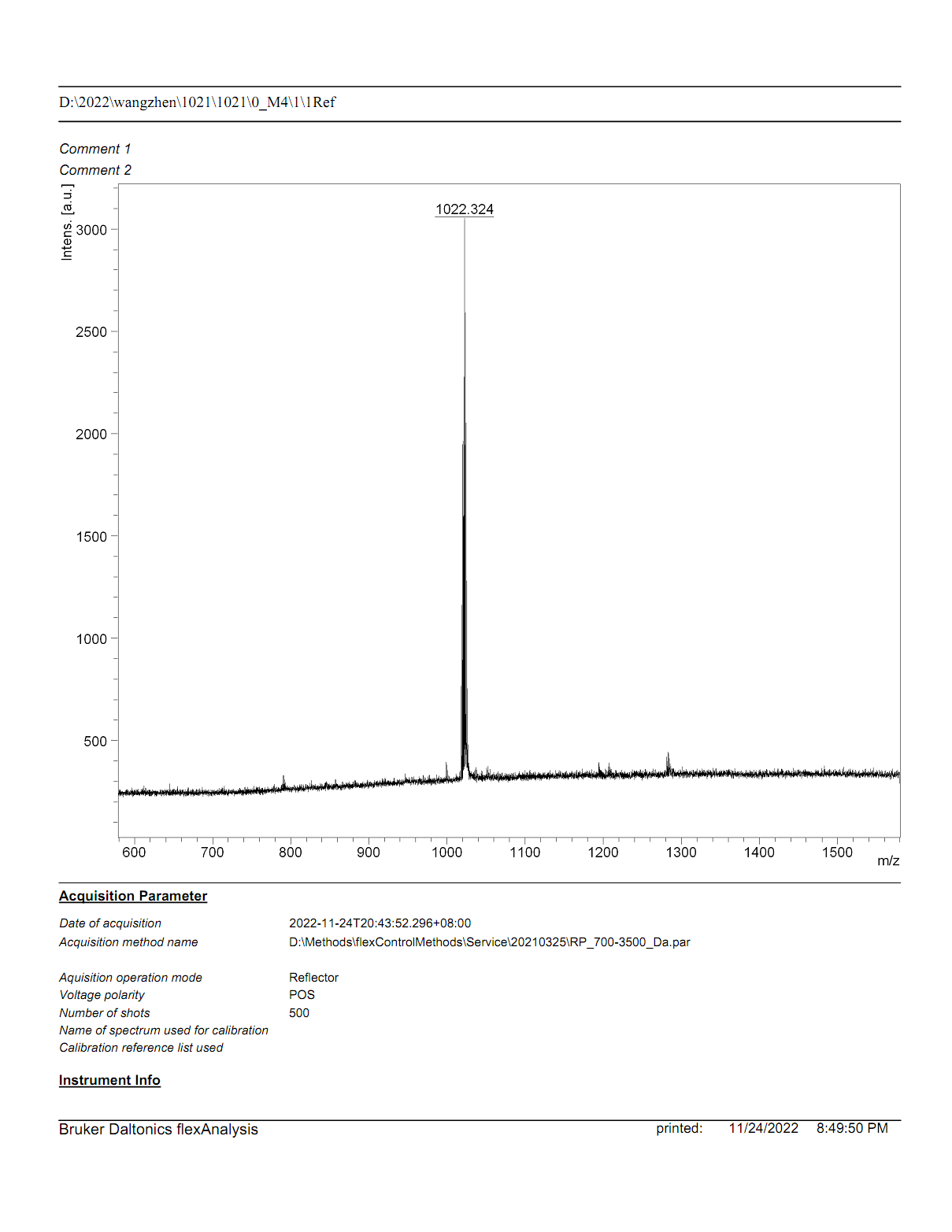


**Figure S2.** MALDI-TOF mass spectrometry of Se-TC.


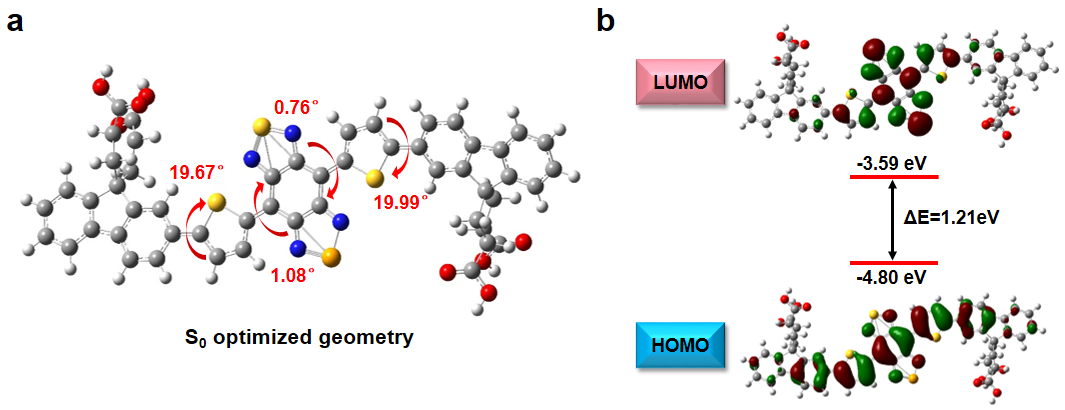


**Figure S3.** (a) The optimized S_0_ geometry, (b) HOMO-LUMO distributions of Se-TC by density functional theory (DFT) calculation using B3LYP/6-31G(d).


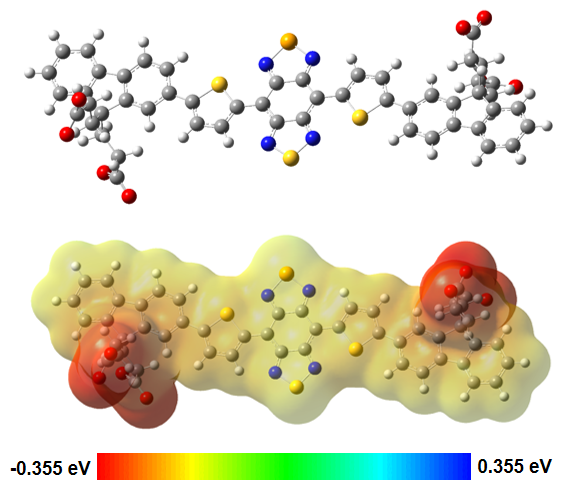


**Figure S4.** Map of electrostatic potential (ESP) surface of Se-TC.


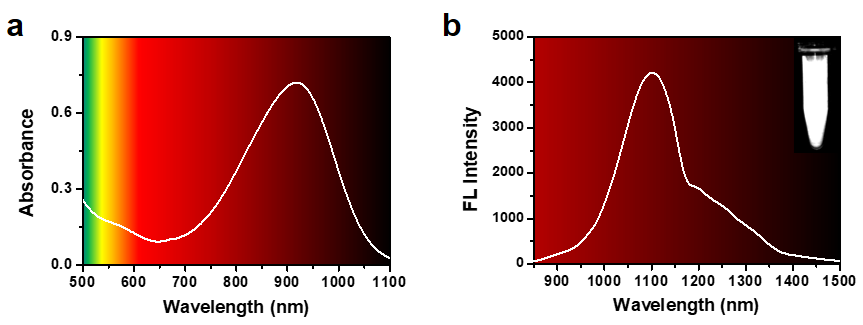


**Figure S5.** The UV–vis absorption spectrum and 808 nm excited fluorescence emission spectrum of Se-TC in tetrahydrofuran (THF).


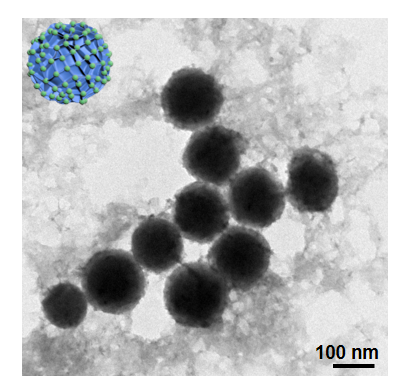


**Figure S6.** The TEM image of self-assembled Se-TC NPs distributed in THF.


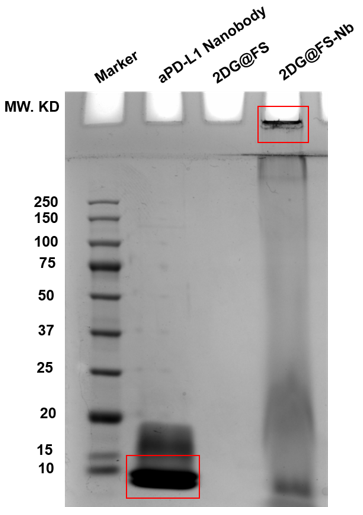


**Figure S7.** SDS-PAGE analysis of aPD-L1 nanobody (Nb), 2DG@FS and 2DG@FS-Nb.


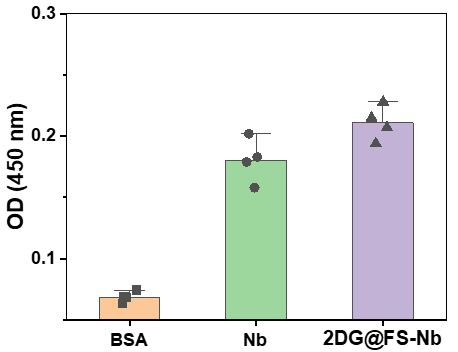


**Figure S8.** The binding activity of aPD-L1 nanobody (Nb) and 2DG@FS-Nb towards PD-L1 antigen.


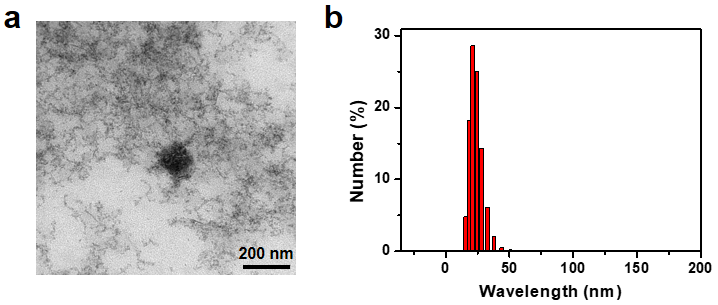


**Figure S9.** (a) TEM image and (b) size distribution of 2DG@FS-Nb at pH 5.5.


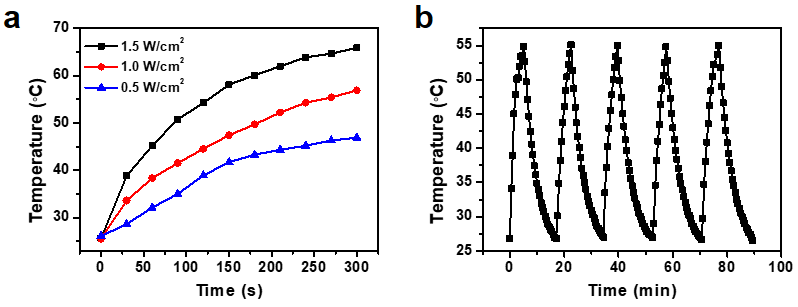


**Figure S10.** (a) Temperature curves of 2DG@FS-Nb aqueous solution under 1064 nm laser irradiation with different power densities. (b) Temperature curve of 2DG@FS-Nb aqueous solution under five consecutive laser on/off cycles (1064 nm, 1 W/cm^2^).


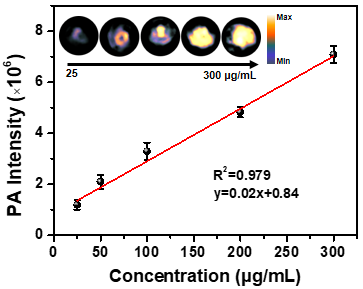


**Figure S11.** The photoacoustic images of 2DG@FS-Nb aqueous solution with different concentrations under 1064 nm laser excitation.


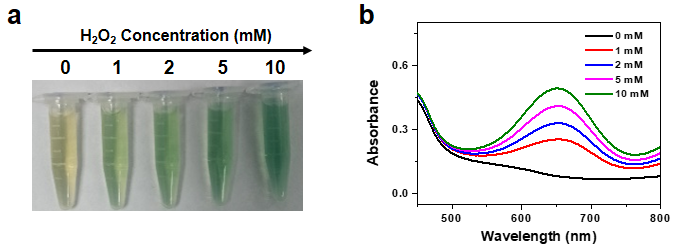


**Figure S12.** (a) The color changes and (b) absorption spectra of the mixed solution of TMB and 2DG@FS-Nb with different concentrations of H_2_O_2_.


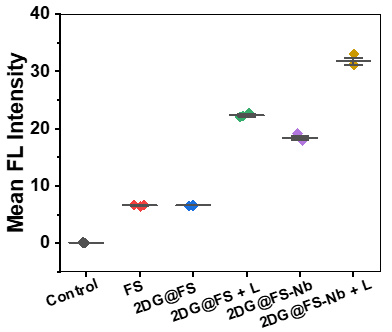


**Figure S13.** The fluorescence intensity analysis of DCF in 4T1 cells with various treatments.


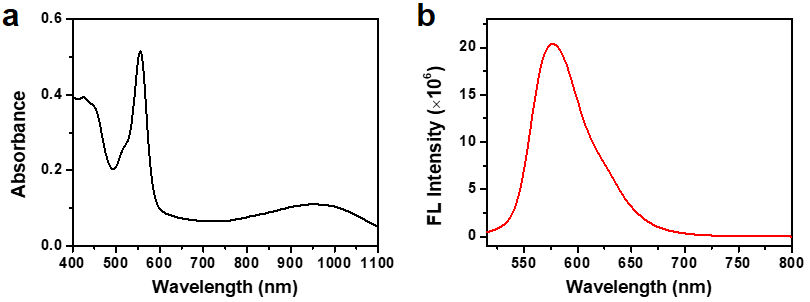


**Figure S14.** (a) UV-vis absorption and (b) fluorescence emission spectra of 2DG@FS-Nb doped with RhB.


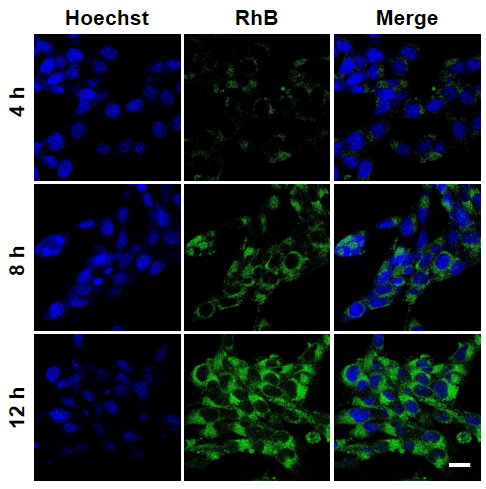


**Figure S15.** Intracellular uptake of 2DG@FS-Nb in 4T1 cells over time. Scale bar: 20 μm.


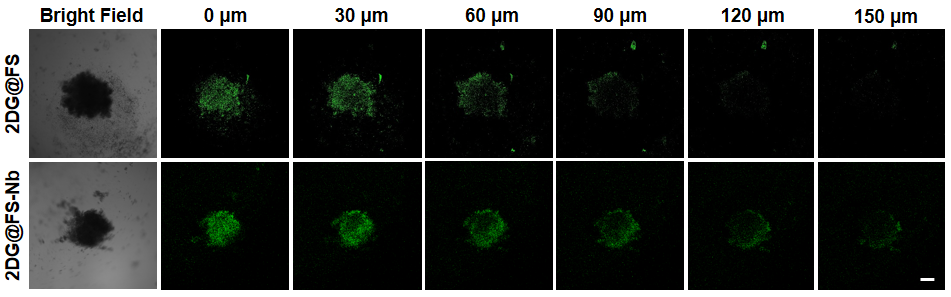


**Figure S16.** The cellular uptake in cell spheroids with different penetration depths. Scale bar: 200 μm.


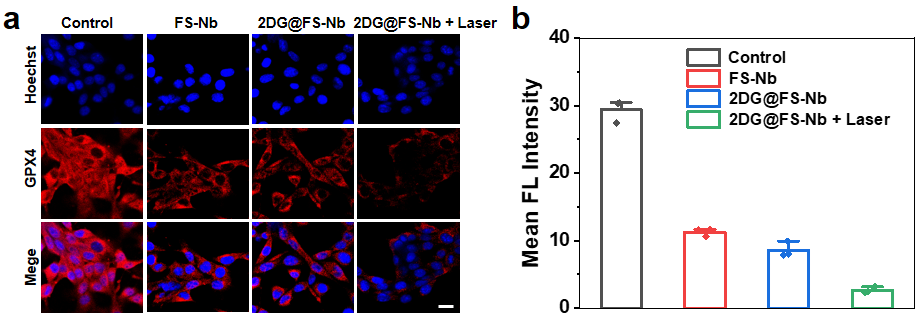


**Figure S17.** (a) Immunofluorescence images and (b) quantitative fluorescence intensity analysis of GPX4 expression in 4T1 cells with various treatments. Scale bar: 20 μm.


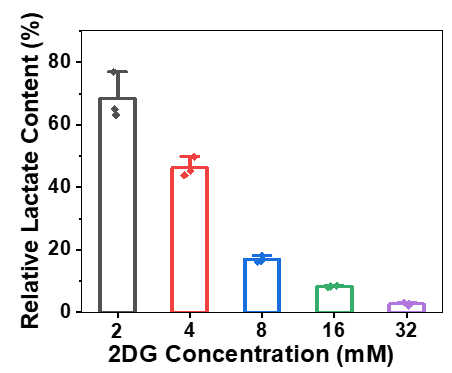


**Figure S18.** Extracellular lactate content of 4T1 cells treated with 2DG@FS-Nb containing different concentrations of 2DG.


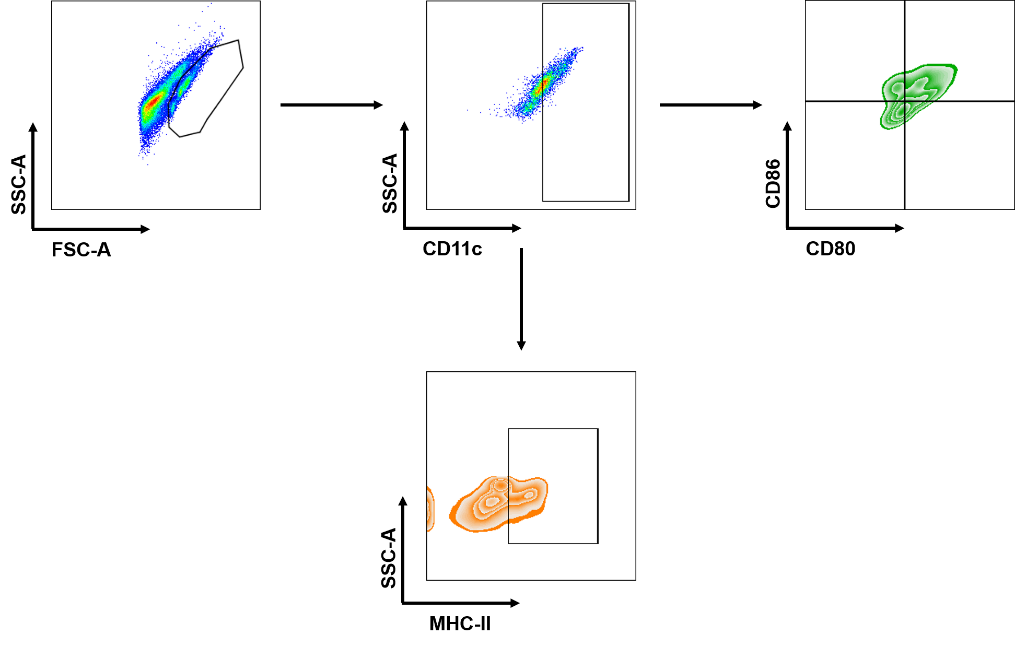


**Figure S19.** The gating strategy used for flow cytometry analysis of DC maturation and MHC-II expression *in vitro*.


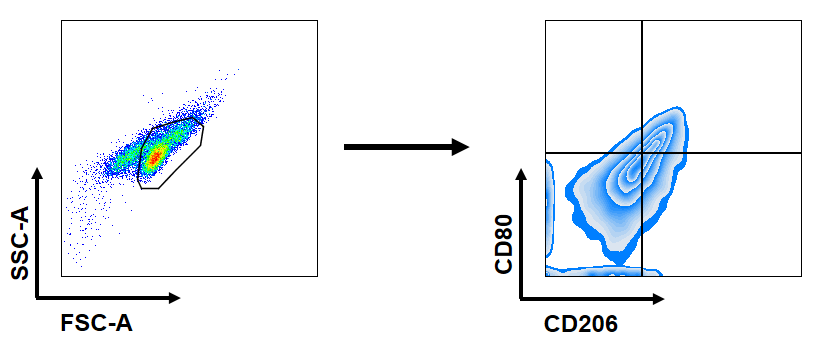


**Figure S20.** The gating strategy of macrophage differentiation after different treatments, CD80^+^CD206^-^ for M1 macrophages and CD80^-^CD206^+^ for M2 macrophages.


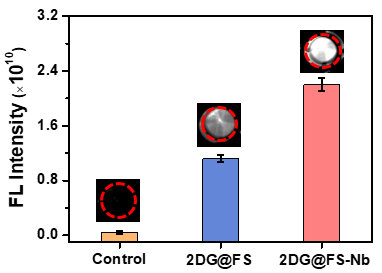


**Figure S21.** NIR-II fluorescence images of 4T1 cells treated with 2DG@FS and 2DG@FS-Nb.


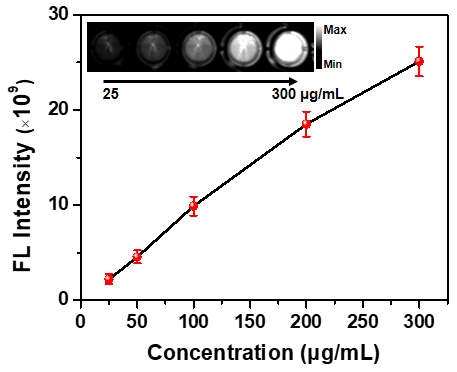


**Figure S22.** NIR-II fluorescence images of 4T1 cells treated with 2DG@FS-Nb with different concentrations.


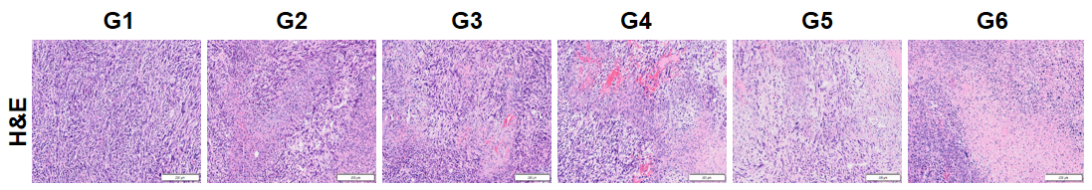


**Figure S23.** H&E staining images of distant tumors with different treatments after 14 days of treatment. Scale bar: 200 μm. G1: PBS, G2: Nb, G3: FS-Nb, G4: 2DG@FS-Nb, G5: FS-Nb + Laser, G6: 2DG@FS-Nb + Laser.


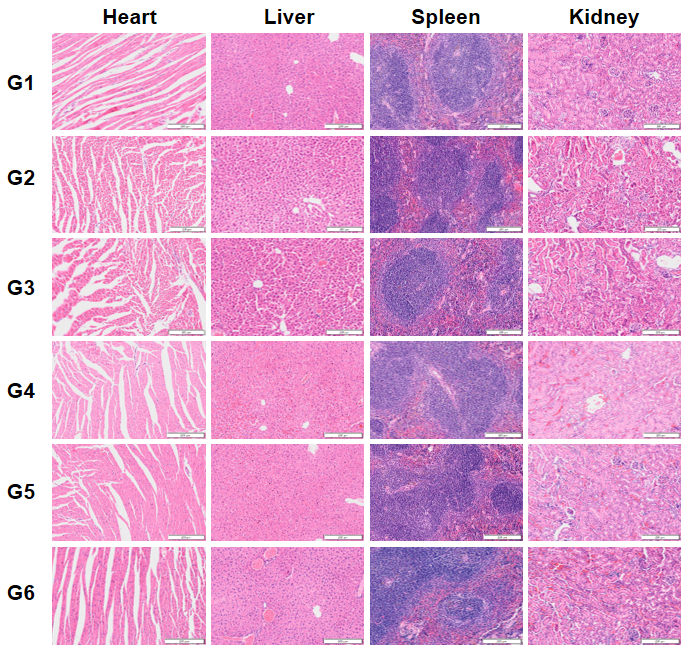


**Figure S24.** H&E staining images of major organs (heart, liver, spleen and kidney) from bilateral 4T1-tumor-bearing mice after various treatments for 14 days. Scale bar: 200 μm. G1: PBS, G2: Nb, G3: FS-Nb, G4: 2DG@FS-Nb, G5: FS-Nb + Laser, G6: 2DG@FS-Nb + Laser.


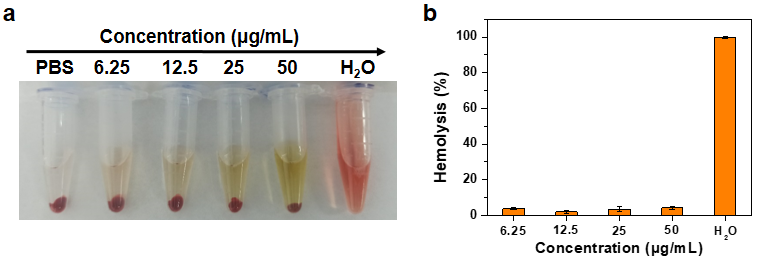


**Figure S25.** (a) Representative hemolysis photographs and (b) quantitative analysis of 2DG@FS-Nb with different concentrations.


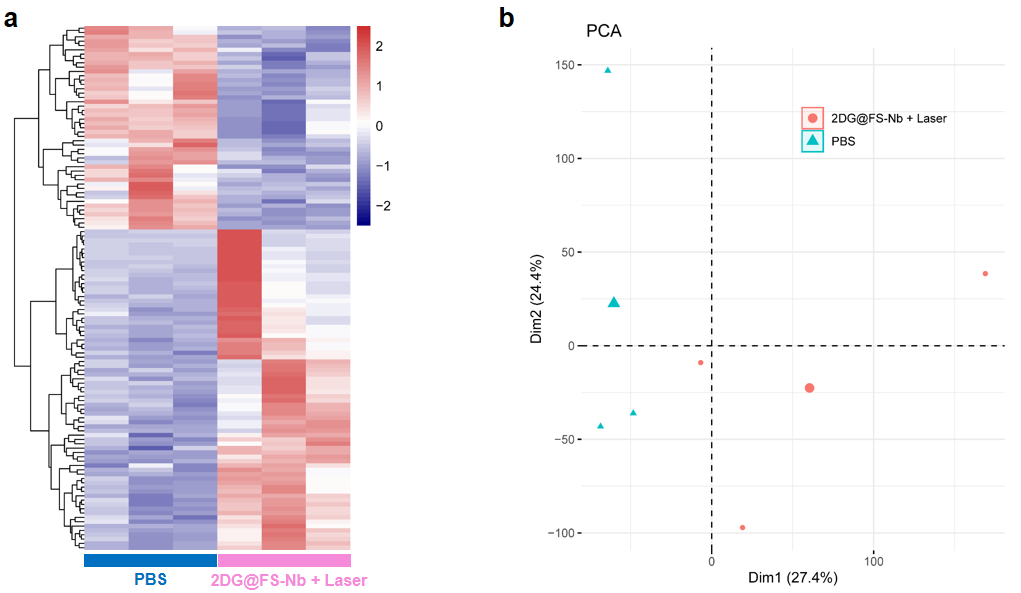


**Figure S26.** (a) Heat map of the DEGs and (b) the graph of PCA between PBS and 2DG@FS-Nb + Laser groups.


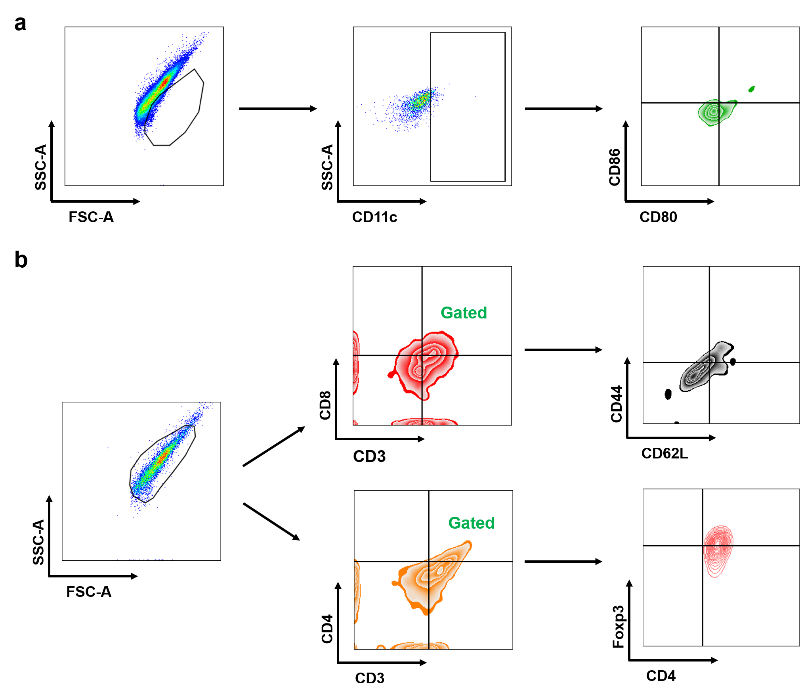


**Figure S27.** The gating strategy of DC maturation, T cell activation, Treg cells in tumor tissues and memory T cells in spleens.


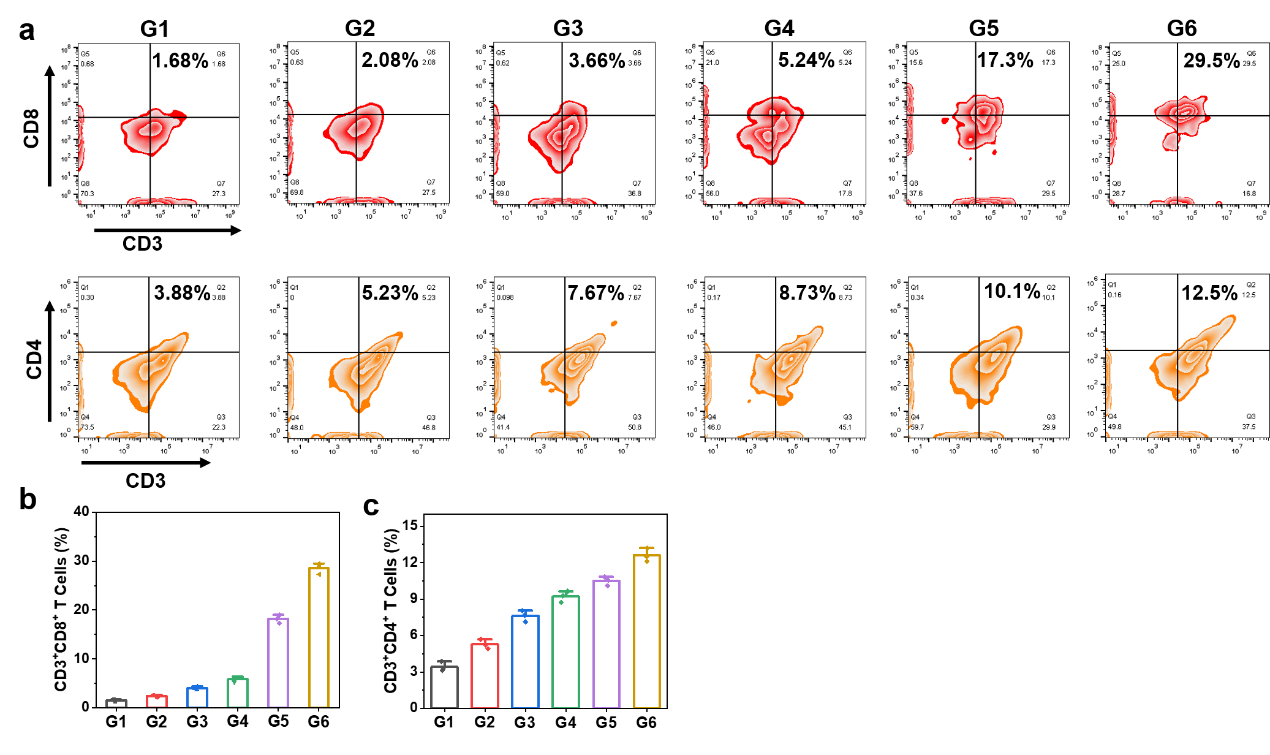


**Figure S28.** (a) Flow cytometry assay and (b) (c) corresponding statistical data of CD3^+^CD8^+^ T cells and CD3^+^CD4^+^ T cells in distant tumor tissues after 7 days of treatment against primary tumors. G1: PBS, G2: Nb, G3: FS-Nb, G4: 2DG@FS-Nb, G5: FS-Nb + Laser, G6: 2DG@FS-Nb + Laser.


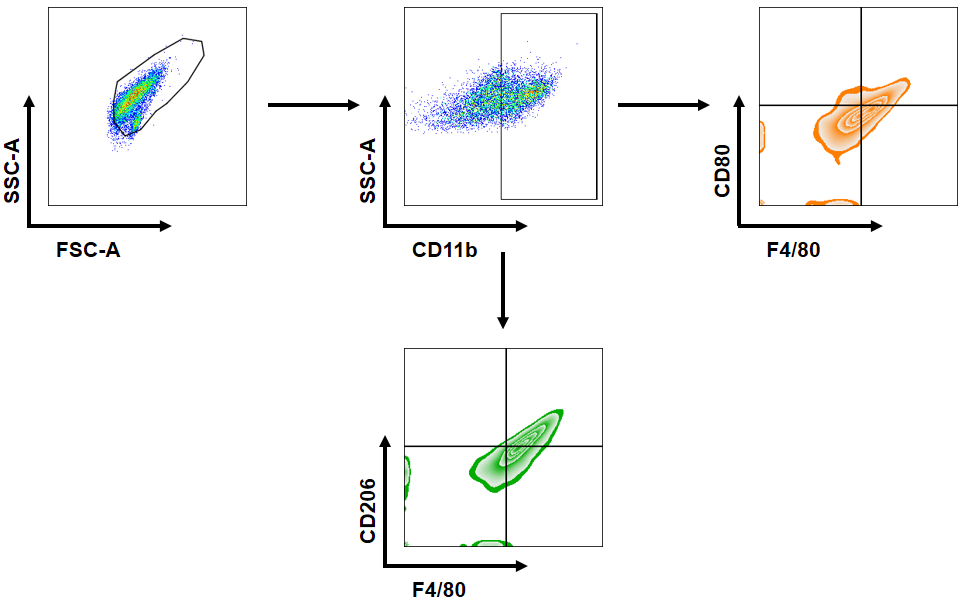


**Figure S29.** The gating strategy of M1 macrophages (F4/80^+^CD80^+^) and M2 macrophages (F4/80^+^CD206^+^) in tumor tissues.


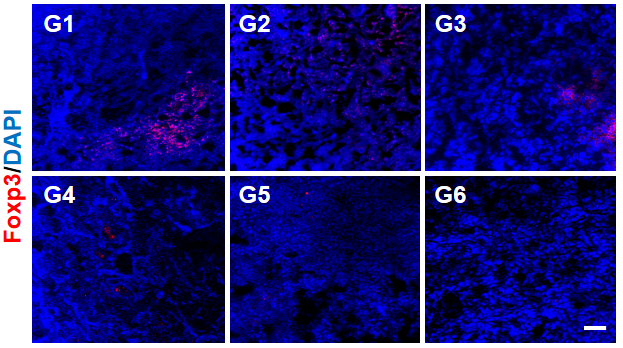


**Figure S30.** Immunofluorescence staining images of Treg cells infiltration in 4T1 tumors with different treatments. Scale bar: 50 μm. G1: PBS, G2: Nb, G3: FS-Nb, G4: 2DG@FS-Nb, G5: FS-Nb + Laser, G6: 2DG@FS-Nb + Laser.


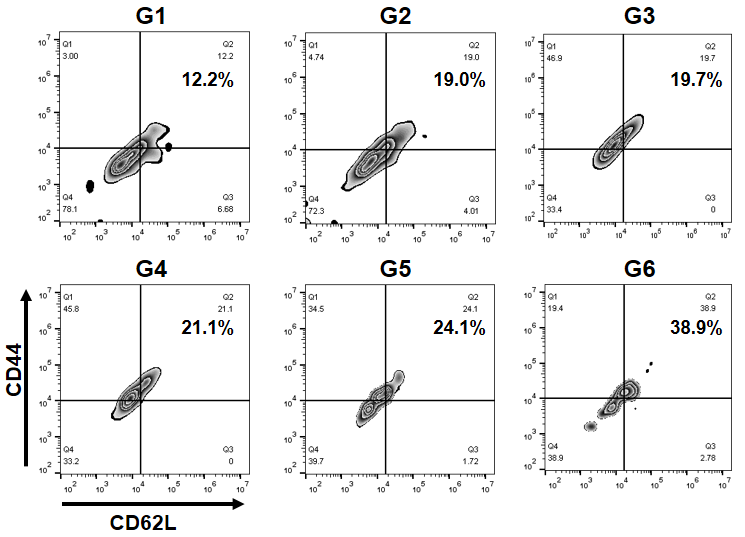


**Figure S31.** Flow cytometric plots of central memory T cells (CD3^+^CD8^+^CD44^+^CD62L^+^) in mouse spleen in various treatment groups. G1: PBS, G2: Nb, G3: FS-Nb, G4: 2DG@FS-Nb, G5: FS-Nb + Laser, G6: 2DG@FS-Nb + Laser.

[1] L. P. Zhu, J. N. Li, Z. Guo, H. F. Kwok, Q. Zhao, *J Nanobiotechnology* **2022**, *20*, 521.

[2] Q. Wang, X. M. Zhang, Y. G. Tang, Y. W. Xiong, X. Wang, C. L. Li, T. X. Xiao, F. Lu, M. Z. Xu, *Pharmaceutics* **2023**, *15*, 2027.

[3] a) J. P. Islamian, M. Hatamian, N. A. Aval, M. R. Rashidi, A. Mesbahi, M. Mohammadzadeh, M. A. Jafarabadi, *Breast* **2017**, *33*, 97; b) Z. Y. Luo, J. N. Xu, J. J. Sun, H. Z. Huang, Z. G. Zhang, W. N. Ma, Z. Y. Wan, Y. W. Y. Liu, A. Pardeshi, S. Li, *Acta Biomater.* **2020**, *105*, 239; c) B. W. Yang, Y. Chen, J. L. Shi, *Angew. Chem., Int. Ed.* **2020**, *132*, 9780.
